# Unveiling Health Security Patterns in the European Union through a Hybrid Entropy-CoCoSo and K-Means Clustering Framework

**DOI:** 10.12688/f1000research.166187.1

**Published:** 2025-06-19

**Authors:** Adel A. Nasser, Yahya Ali Al-Samawi, Abed Saif Ahmed Alghawli, Amani A. K. Essayed

**Affiliations:** 1 Department of Information Systems and Computer Science, Sa’adah University, Sa’adah University, Sa’adah, Yemen; 2 Department of Artiﬁcial Intelligence, Modern Specialized University, Sana'a, Yemen; 3 Information Technology, Modern Specialized University, Sana'a, Yemen; 4 Department of Computer Science, College of Sciences and Humanities, Prince Sattam bin Abdulaziz University, Al Kharj, Riyadh Province, 16700, Saudi Arabia

**Keywords:** health security, European Union, multi-criteria decision-making, entropy, combined compromise solution, clustering, ranking

## Abstract

**Objectives:**

This study aimed to examine health security (HeS) patterns across European Union (EU) member states to address intra-regional disparities in health security, align with EU-wide policy objectives, and propose evidence-based recommendations for harmonizing preparedness measures while respecting national sovereignty

**Methods:**

This research employed a hybrid multi-criteria decision-making framework, combining the Entropy Weight Method and Combined Compromise Solution (CoCoSo), to assess and rank EU countries, drawing on six Global Health Security Index indicators. K-means clustering classified countries into three performance tiers: High, Intermediate, and Dangerous. Data from the GHSI (2019, 2021) and the aggregated 2017–2021 period were analyzed to track temporal trends and cross-regional performance disparities. A comparative analysis of HeS priorities with the African and Eastern Mediterranean (EMR) Regions further contextualized the EU's HeS landscape.

**Results:**

Detection and Reporting, and Rapid Response emerged as the most critical dimensions influencing performance, while Risk Environment and Compliance with Norms showed minimal differentiation. High-performing countries, such as Finland and Germany, demonstrated resilience in surveillance and rapid response, while lower-tier nations, Cyprus, Luxembourg, Malta, and Romania, exhibited systemic vulnerabilities in biosecurity and emergency planning. Post-2019, health system resilience gained prominence, while compliance and risk environment remained neglected. The temporal analysis highlighted post-pandemic shifts in health system disparities. Cross-regional comparisons underscoring context-specific challenges.

**Conclusion:**

This study highlights the need for targeted investments in surveillance systems, laboratory infrastructure, and crisis preparedness to address specific gaps in different clusters. A metrics-driven framework can reduce regional disparities, promoting equity in preparedness. Policymakers should adopt a collaborative approach to mitigate crises, using high-performing clusters as benchmarks.

**Glossary**
EU (European Union): An alliance of 27 European countries focused on shared economic and governance goals, including collaborative efforts to strengthen health security systems and manage emergencies.
EMR (Eastern Mediterranean Region): A World Health Organization (WHO) regional classification covering 22 countries in the Middle East, North Africa, and parts of Asia.
CoCOSo (Combined Compromise Solution): A multi-criteria decision-making method that integrates multiple aggregation strategies to rank alternatives
Entropy: Entropy Weight Approach
GHSI: Global Health Security Index – A comprehensive assessment of a country's health security capabilities
MCDM: Multi-Criteria Decision-Making
HeS: Health security

## 1. Introduction

Health security (HeS) is a dynamic framework designed to protect populations from both known and emerging health threats. Expanding beyond traditional public health models, HeS integrates proactive disease surveillance, emergency preparedness, and responsive outbreak management while fostering international collaboration and technological innovation for early risk detection and mitigation. ^1^ Its multi-faceted strategy addresses current challenges while anticipating evolving global health risks.

Significant shortcomings in global preparedness for health emergencies were exposed during the COVID-19 crisis, underscoring the urgent need for resilient frameworks that harmonize surveillance, rapid response mechanisms, and equitable resource allocation. ^2– 4^ Health security demands continuous adaptation to shifting threats, ^5^ sustained by iterative monitoring, flexible governance, and prioritized benchmarks to evaluate systems, guide investments, and address vulnerabilities. ^5– 7^ However, resource constraints and conflicting national priorities complicate alignment, necessitating multilateral cooperation to optimize regional collaboration and resource allocation. ^6, 8^

The Global Health Security Index (GHSI), a joint initiative of the Nuclear Threat Initiative and Johns Hopkins Center for Health Security, measures pandemic preparedness in 195 countries by evaluating performance in six core categories: disease prevention, outbreak detection, crisis response, healthcare system robustness, compliance with global standards, and environmental and societal risk conditions. ^9^ Despite its global adoption, critics highlight critical flaws in its methodology ^5, 6, 10^: (1) A rigid scoring system that uniformly weights criteria, ignoring regional disparities; (2) Insufficient granularity for regional and sub-regional ranking, clustering, or dynamic trend analysis; and (3) Unable to capture evolving performance, particularly pre- and post-COVID-19 weightings, rankings, and clustering shifts. These shortcomings significantly constrain the GHSI’s effectiveness as a tool for context-aware, policy-relevant analysis.

Employing hybrid multi-method models—such as entropy-TOPSIS-K-means in Africa, ^5^ entropy-VIKOR-K-means in the Eastern Mediterranean Region (EMR), ^6^ and D-CRITIC-CoCoSo-K-means in Western Asia ^10^—has demonstrated significant value in health security evaluation. These approaches combine objective weighting, robust ranking, and clustering techniques to enable nuanced performance analysis, priority identification, and pattern exploration across diverse national contexts. By sequentially applying these methods, hybrid frameworks offering a more rigorous, interpretable, and context-sensitive foundation for decision-making. ^5, 6, 10^ However, despite their proven effectiveness in other global regions, health security analyses within the EU still lack hybrid frameworks—particularly those tailored to the region’s distinct health dynamics and policy context.

Global indices like the GHSI homogenize EU member states within international rankings, overlooking the bloc’s distinct supranational governance structures, such as the European Health Union (EHU). While the EHU mandates cross-border collaboration, its implementation is often fragmented. Unlike regional studies in Africa, ^5^ the EMR, ^6^ or Western Asia ^10^—which prioritize context-specific challenges like zoonotic spillovers or conflict-driven infrastructure deficits—EU disparities largely stem from uneven policy adoption and variable healthcare resilience. For instance, Forman and Mossialos critique the EU’s lack of harmonized surveillance, ^11^ and Rees et al. (2024) ^12^ highlight operational inefficiencies in pandemic response.

Parallel research stresses the centrality of resilient health systems and supply chain redundancies in crisis resilience, particularly in mitigating disruptions during acute shocks. ^13, 14^ Expanding the discourse, study ^15^ highlight persistent zoonotic spillover risks, urging preemptive One Health strategies to address interspecies disease transmission. Complementary analyses reinforce calls for integrated, data-centric approaches to health security (HeS). Brown et al. (2022) conducted a systematic review of health system-security linkages, revealing a persistent disconnect between emergency preparedness frameworks and routine healthcare operations. ^16^ Their findings support the integration of HeS objectives—such as outbreak response protocols—into core health system functions, rather than maintaining them as discrete initiatives. Furthermore, their analysis highlights the need for precise policies that tackle both overarching healthcare deficiencies and the specific contextual factors within EU member states.

Region-specific challenges are further elucidated by El Samad et al. (2022), who document resource constraints, demographic shifts, and chronic disease burdens in Mediterranean EU states, proposing data-driven optimization of healthcare delivery to address these pressures. ^17^ Similarly, Rees et al. (2024) critique heterogeneous pandemic responses across Europe, arguing that operational inefficiencies stem not from a lack of technical systems but from underdeveloped coordination and adaptability mechanisms. ^12^ These findings collectively underscore the critical need for health security investigations that are specifically tailored to the EU context, accounting for its unique policy landscape, and regional disparities. A failure to do so risks misinterpreting the underlying dynamics and hindering the development of effective, context-appropriate solutions. ^6, 10^

**Research problem and questions:** Existing EU-focused studies lack longitudinal, hybrid multi-method analyses that effectively capture the region’s unique supranational governance structure and address intra-regional disparities in health security policy implementation and resilience. To bridge this gap, this study explores:

- How do health security priorities in the EU—such as detection and rapid response—differ from those in non-EU regions (e.g., Africa’s prevention focus, EMR’s health system emphasis)?
- What clusters of performance exist among EU member states (2017–2021), and how do these reflect evolving post-pandemic challenges like infrastructure gaps or surveillance fragmentation?
- How can supranational bodies leverage ranking and cluster-tiered findings to harmonize preparedness while respecting national autonomy?

**Research objectives:** This research seeks to assess and categorize health security preparedness among European Union (EU) member states by using a hybrid Entropy-CoCoSo and K-Means clustering approach. By identifying key performance indicators and clustering patterns, the research seeks to address intra-regional disparities in health security, align with EU-wide policy objectives, and offer actionable recommendations for harmonizing preparedness measures while respecting national autonomy.

Aligned with the EU’s 2017–2021 health security trajectory—spanning pre-pandemic benchmarks under the Joint Action on Health Security to post-COVID-19 reforms like the Health Emergency Preparedness and Response Authority (HERA)—this analysis informs actionable strategies. By identifying underperforming clusters (e.g., Cyprus, Malta), the findings support HERA’s mandate to allocate infrastructure funding and standardize cross-border crisis protocols. Concurrently, the prioritization of detection and rapid response aligns with the EHU’s 2023–2027 strategy to unify surveillance systems, offering a metrics-driven blueprint for reconciling implementation gaps.

**Scientific contribution and implications:** To overcome the limitations of global indices and provide a more granular assessment of EU health security, this study makes several key contributions to the field of health security. It improves research methods by using a combination of Entropy-CoCoSo and K-means clustering to evaluate health security in European Union (EU) member states—something that hasn't been done in this area before. This method expands the body of scientific modeling methodologies applicable to real-world multi-criteria problems, showcasing how hybrid decision-making and clustering techniques can generate actionable insights for evaluating and strengthening health system resilience. By integrating concepts from multi-criteria decision-making (MCDM) and machine learning, the study fosters interdisciplinary innovation, enabling the fusion of theoretical and computational approaches to tackle complex public health challenges.

Furthermore, this research promotes goal-oriented science by enabling precise monitoring, evaluation, and acceleration of progress on global health security (GHS) goals. Unlike static global indices like the Global Health Security Index (GHSI), which often overlook regional nuances and temporal dynamics, the used model offers a granular, dynamic, and context-sensitive assessment. It identifies intra-regional disparities and temporal shifts in health security priorities, providing a replicable, scalable framework that supports tailored policy guidance and resource allocation at both national and supranational levels.

The study also contributes to a more equitable and effective global health dialogue by highlighting divergent regional priorities between the EU, Africa, and the Eastern Mediterranean Region (EMR). These findings emphasize the need for context-specific policies and the rejection of one-size-fits-all strategies. By recognizing regional heterogeneity in health security drivers—such as detection in the EU, prevention in Africa, and health system robustness in the EMR—the study encourages localized policy frameworks and fosters more equitable partnerships. It also enables donor agencies and multilateral institutions to realign programs with the unique health security realities and development priorities of each region.

Additionally, the study maps EU health security priorities and classifies member states into performance-based clusters over the 2017–2021 period, revealing systemic disparities and resilience gaps exposed by the COVID-19 pandemic. This cluster-based assessment facilitates differentiated interventions that respect national autonomy while promoting strategic harmonization through bodies like the European Health Union (EHU) and the Health Emergency Preparedness and Response Authority (HERA). By offering empirical insights and data-driven recommendations, the study equips policymakers with the tools to design forward-looking, region-specific responses to evolving health threats. Finally, the analytical framework established here serves as a foundation for future research, including cross-regional comparisons and subnational analyses, supporting a globally adaptable model for health security evaluation.

The remainder of this paper is structured as follows: Section 2 details the hybrid Entropy-CoCoSo-K-means methodology, explaining entropy-based criterion weighting, CoCoSo’s ranking, and K-means clustering for dynamic tier classification. Section 3 evaluates EU health security performance through cross-regional benchmarks (Africa, EMR) and temporal analysis of intra-EU clusters (2019–2021). Section 4 analyzes trends, policy gaps, and actionable recommendations for stakeholders. Section 5 concludes with strategic interventions and future research directions.

## 2. Methods

Multi-Criteria Decision-Making (MCDM) methods have become essential tools across disciplines such as health security, sustainability, tourism, healthcare, engineering, and financial risk management. MCDM has guided strategic planning in various fields—such as prioritizing eco-hotel performance, ^18, 19^ comparing hospital health literacy levels, ^20^ and managing information security in banks. ^21^ It has also supported healthcare resource allocation and optimization. ^22^ Globally, MCDM has played a vital role in addressing post-COVID-19 challenges, with applications in vaccine selection, biomedical decision-making, health security evaluation, and sustainability benchmarking.

In sustainability, entropy-VIKOR and related models have been used to compare national strategies. ^23, 24^ Hybrid MCDM models—such as Entropy-CoCoSo and EDAS—have ranked biomedical materials, ^25^ evaluated COVID-19 vaccine options, ^26^ and assessed urban stress levels. ^27^ In regional health security studies, integrated MCDM and clustering approaches have been employed to evaluate disparities. ^5, 6, 10^ Furthermore, clustering tools such as K-means enhance the scalability of MCDM by enabling the analysis of complex, multidimensional datasets. ^5, 6, 10, 28, 29^

Health security assessment can be formulated as a complex Multi-Criteria Decision-Making (MCDM) problem, characterized by dynamic, interconnected, and evolving dimensions that demand robust evaluation frameworks. The need to assess health security improvements over multiple years adds temporal complexity, requiring the consideration of shifting regional trajectories and the comparative prioritization of diverse global settings. ^10^ Health security itself encompasses a multidimensional network of interdependent targets—such as prevention, detection, response, governance, and international collaboration—each varying in criticality depending on regional vulnerabilities and temporal factors. ^9^ Prioritizing these targets across regions involves navigating competing criteria, dynamic weightings, and complex trade-offs. Moreover, geographic priority analysis, as another MCDM layer, is critical for systematically identifying systemic weaknesses within regional health security infrastructures and informing the strategic direction of future interventions and research. ^6^ Thus, solving health security assessment as an MCDM problem requires hybrid, adaptive methodologies capable of integrating heterogeneous, time-sensitive, and interdependent criteria for dynamic and actionable policy development. ^5, 6, 10^

Moreover, objective weighting methods, such as Multi-Criteria Decision-Making (MCDM) techniques, offer robust solutions by assigning adaptable, data-informed weights to health security indicators, reflecting their changing significance over time. ^10^ In MCDM, objective weighting methods facilitate the nuanced ranking and categorization of indicators based on their importance, ^21, 30– 32^ allowing comparisons across different regions and periods and highlighting those areas most in need of improvement. ^6, 10^

By determining indicator importance for specific regions, these techniques enable tailored strategies and interventions that address each region's unique needs and challenges more effectively, allow comparisons between regions, and help identify trends and changes over time. This facilitates more accurate forecasting and long-term planning and promotes a better understanding of regional disparities and potential areas for improvement. ^10^

Through indicator categorization, resources can be allocated more efficiently based on the relative importance of different factors. ^5, 22^ Moreover, the ability to reveal hidden patterns in indicator importance across regions and time can lead to new insights and research directions that might otherwise be overlooked. ^6^

Furthermore, combining hybrid weighting with ranking MCDM methods and clustering techniques facilitates the detailed categorization of countries based on comprehensive performance profiles, spotlighting those most urgently needing enhancement. ^5, 23, 24, 33, 34^ Integrating dynamic analyses further enables continuous tracking of progress and emerging trends, allowing policymakers to make evidence-based decisions that respond flexibly to the evolving landscape of HeS development. ^5, 6, 10^

These methods enable a more holistic evaluation of health security by considering multiple criteria simultaneously. ^5^ Comparing performance across different time frames allows for the tracking of progress or regression in health security measures over time. ^6^ Grouping countries by region facilitates the identification of regional trends, strengths, and weaknesses, informing policymakers about areas requiring improvement and helping prioritize resource allocation. ^10^ Countries can also use these rankings to benchmark their performance against peers and identify best practices. Furthermore, highlighting disparities in health security can encourage global collaboration and support for underperforming countries, increase public awareness of health security issues, and potentially drive citizen engagement. ^5^ Additionally, the results can guide further research into factors influencing health security performance.

In regional MCDM applications across various domains, hybrid models have demonstrated high effectiveness. For example, an Entropy-VIKOR hybrid was successfully applied to select dental restorative materials, demonstrating the method’s capability to rank alternatives effectively in biomedical contexts. ^25^ Similarly, an Entropy-EDAS integration was employed for diesel engine parameter optimization, validated by other methods like WASPAS and TOPSIS. ^35^

In the field of health security, MCDM methods have also shown strong applicability. TOPSIS and VIKOR were used to evaluate COVID-19 vaccine selection strategies, considering factors such as safety, cost, and implementation challenges. ^26^ A notable application during the COVID-19 era is seen in a study on Western Asia, where a D-CRITIC-based MCDM model used the CoCoSo method for ranking countries, followed by K-means clustering to effectively identify varying pandemic readiness levels across nations. ^10^ Similarly, in Africa and the Eastern Mediterranean Region (EMR), studies combined entropy weighting with TOPSIS and VIKOR models, respectively, alongside K-means clustering to categorize countries by their health security performance. ^5, 6^ Additionally, a CRITIC-TOPSIS model was used to assess stress levels in major Indian cities during the COVID-19 outbreak, showing that urban overload and vulnerability could be systematically measured with MCDM techniques. ^27^

The Entropy-CoCoSo approach has been widely applied in diverse fields, including engineering sustainability selection ^36^ and spatiotemporal analysis of business environments. ^33^ These studies collectively demonstrate that MCDM approaches—especially hybrid models integrating entropy, CRITIC, D-CRITIC, TOPSIS, VIKOR, CoCoSo, EDAS, WASPAS, and K-means clustering—have become essential for accurately assessing health security status, identifying risk priorities, and guiding targeted policy interventions across different geographic and socio-economic contexts.

Each MCDM method, however, has its own advantages and disadvantages depending on the problem at hand, the nature of available data, and decision-maker preferences. Different MCDA methods may produce conflicting recommendations, leading to potential uncertainty and confusion. Furthermore, they often require distinct types of input data, weighting schemes, and assumptions.

In this study, we used the entropy method to address the first research question: determining health security priorities in the EU and comparing them with those derived for Africa. ^5, 6^ This decision is based on several key considerations. First, the entropy method is one of the most popular objective weighting techniques for determining criteria weights, widely recognized for its ability to measure variability within data, reduce subjective bias, ensure impartiality, and offer adaptability across diverse fields, including healthcare and health security decision-making. Second, the choice aligns with consistency across comparative studies, as both the African and Eastern Mediterranean Region (EMR) analyses used the entropy method, facilitating a more coherent and valid cross-regional comparison.

For the ranking process, we selected the CoCoSo method due to its robustness. CoCoSo ranks alternatives by integrating weighted sum, weighted product, and exponential compromise models, thus minimizing the bias that can result from relying on a single aggregation technique. However, it is important to acknowledge a limitation: the Entropy-CoCoSo model has not yet been widely applied to Global Health Security Index (GHSI)-related frameworks, particularly in combination with entropy weighting. This lack of prior application introduces some uncertainty regarding its comparative performance and interpretability relative to more established methods such as TOPSIS, VIKOR, EDAS, and WASPAS. Finally, for clustering, we selected K-means due to its proven effectiveness in previous health security MCDM studies for grouping countries by performance profiles, enabling clearer identification of regional trends and risk priorities.

Thus, the combined Entropy-CoCoSo-K-means approach offers a comprehensive, objective, and innovative methodology for our multi-year evaluation of health security across the EU. Our analysis adhered to a structured workflow comprising five sequential stages. Below, we outline the rationale and execution of each phase.

### 2.1 Health security indicators and data source integration

This study utilizes the Global Health Security Index (GHSI) datasets from 2019 and 2021, ^37^ developed by the Johns Hopkins Center for Health Security, to evaluate health security performance across EU member states based on six core criteria: Prevention (PRE), Detection and Reporting (D&R), Rapid Response (RRe), Health System (HSy), Adherence to International Norms (AIN), and Contextual Risk Environment Factors (REF). The GHSI is a robust, internationally recognized tool designed to assess countries’ capacities to prevent, detect, and respond to public health threats. Its methodological rigor and comprehensive framework make it uniquely suited for evaluating pandemic preparedness. ^5, 6, 9, 10^

One key advantage of the GHSI lies in its global standardization, enabling consistent cross-national comparisons using validated, peer-reviewed indicators. ^9^ This ensures that health security performance is measured through universally accepted criteria grounded in international public health frameworks rather than arbitrary or country-specific metrics. Moreover, the GHSI’s scientific foundation and widespread international adoption as a benchmark for health security have been demonstrated through numerous prior studies conducted across various geographic contexts, further supporting its credibility and relevance.

In recognition of this, researchers have conducted statistical analyses of GHSI metrics alongside in-depth evaluations of related policy measures and organizational frameworks. ^38, 39^ Multi-criteria decision-making (MCDM) and machine learning-based investigations leveraging the GHSI have been applied effectively in Africa, ^5^ the Eastern Mediterranean Region (EMR), ^6^ and Western Asia. ^10^ These applications highlight the GHSI’s adaptability and reliability as a benchmarking tool across diverse health and governance environments, reinforcing its suitability for the objectives of this study.

Taking into account the significance of the GHSI as a tool for health security, and recognizing that one of the primary objectives of this study is to compare weighting across regions, the performance metrics for the main six GHSI indicators were selected as the primary means to assess the weights of the indicators, as well as to rank and cluster EU countries. This study utilized three performance metrics for the years 2019 and 2021, along with the average scores from 2017 to 2021, to analyze the weights, ranking, and clustering of EU countries.

As stated previously, this study aims not only to analyze health security priorities within the EU but also to compare the entropy weighting results with prior regional research from the African region ^5^ and the Eastern Mediterranean Region (EMR). ^6^ Previous studies calculated weights based solely on the data from 2019 and 2021, without considering the entire time period from 2017 to 2021. Therefore, this study further utilized three performance metrics for the years 2019 and 2021, along with the average scores from 2017 to 2021, to analyze the weights among each of the regions: the African region and the Eastern Mediterranean Region (EMR).

### 2.2 Quantifying regional disparities in health security priorities in EU region: An entropy-based assessment of factor significance

The Entropy Method, grounded in information theory, ^40^ objectively assigns criterion weights in multi-criteria decision-making (MCDM) by analyzing data variability. ^22^ This approach quantifies the informational value of each criterion through entropy calculations, prioritizing those with higher divergence (i.e., lower entropy) to minimize subjective bias. ^41^ By algorithmically deriving weights from empirical data distributions, it ensures transparency, scalability, and adaptability, making it ideal for diverse fields. ^5, 6, 22, 41^ While reliant on robust data quality, its mathematical rigor supports reproducible outcomes and complements expert judgment, bridging data-driven objectivity with domain-specific insights in complex decision scenarios. ^5, 6^

Following the framework outlined in, ^5, 6^ the process begins by constructing a decision matrix (G), as shown in Equation (1), which organizes health security ratings for each country and indicator. Here, G represents the decision matrix, where $g_{\mathrm{ij}}\;$ denotes the health security rating of country i for indicator j. In this matrix, *m* denotes the number of nations analyzed, while *n* corresponds to the quantity of health security indicators assessed.

$$G=\begin{array}{cccc} g_{11} & g_{12} & \ldots & g_{1n}\\ \ldots & ... & \ldots & \ldots \\ g_{m1} & g_{m2} & \ldots & g_{\mathrm{mn}} \end{array},$$ (1)

Next, to ensure comparability across heterogeneous criteria, each $g_{\mathrm{ij}}\;$ element is normalized using Equation (2).

$$n_{\mathrm{ij}}=\frac{g_{\mathrm{ij}}}{\sum_{i=1}^{m}g_{\mathrm{ij}}}.$$ (2)

Following this, Equation (3) calculates the entropy value ${\mathrm{Ent}}_{j}\;$ for each indicator j, quantifying the uncertainty or spread in the dataset.

$${\mathrm{Ent}}_{j}=-\left(\frac{1}{\ln\left(m\right)}\right)\sum_{i=1}^{m}n_{\mathrm{ij}}*\ln\;n_{\mathrm{ij}},\forall j.$$ (3)

The final step involves calculating divergence and assigning criterion weights. Divergence, derived as $1-{\mathrm{Ent}}_{j}$ , reflects the informational significance of each indicator. This value is normalized to produce the final weights $W_{j}$ , as formalized in Equation (4). Criteria with higher variability—indicating greater discriminatory power—receive elevated weights, prioritizing them in the decision-making process.

$$W_{j}=\frac{1-{\mathrm{Ent}}_{j}}{\sum_{j=1}^{n}1-{\mathrm{Ent}}_{j}},\forall j.$$ (4)

For the EU-2019, as an example, a decision matrix ( Table 1) was structured with performance scores for 27 EU countries across predefined HeS indicators. Next, normalization using Equation (2) standardized the data ( Table 2), scaling values relative to column sums. Entropy values ( Equation 3, Table 3) were then computed to quantify each indicator’s informational uncertainty. Divergence scores, calculated via Equation (4), translated entropy into relative importance, with higher divergence indicating greater criterion utility. Finally, normalized divergence values yielded definitive weights, prioritizing indicators with higher variability to ensure data-driven, unbiased policy recommendations. Detailed calculations for this step are available in the supplementary file (Step 2- sheet 2.1(a,b,c)). ^42^

**Table 1.** HeS decision matrix for EU (2019).

N	Country	Prevention	Detection and reporting	Rapid response	Health system	Compliance with norms	Risk environment
**1**	Austria	53.3	38.8	47.9	54	63.9	86.5
**2**	Belgium	57.5	52.9	57.5	64.3	60.6	78.4
**3**	Bulgaria	66.7	61.7	49	58.3	69.4	63.5
**4**	Croatia	51.3	37.8	37	51.4	55	66.2
. **.** **.**	. **.** **.**	. **.** **.**	. **.** **.**	. **.** **.**	. **.** **.**	. **.** **.**	. **.** **.**
**27**	Sweden	80.6	64.6	46.1	53.6	69.4	83.8

Table 1 displays the health security (HeS) performance scores for 27 EU countries across six key indicators: Prevention, Detection and Reporting, Rapid Response, Health System, Compliance with Norms, and Risk Environment for the year 2019.

**Table 2.** Standardized HeS scores for EU (2019).

N	Country	Prevention	Detection and reporting	Rapid response	Health system	Compliance with norms	Risk environment
**1**	Austria	0.038	0.030	0.033	0.038	0.038	0.043
**2**	Belgium	0.041	0.040	0.039	0.046	0.036	0.039
**3**	Bulgaria	0.047	0.047	0.034	0.041	0.042	0.032
**4**	Croatia	0.036	0.029	0.025	0.036	0.033	0.033
**.** **.** **.**	. . .	. . .	. . .	. . .	. . .	. . .	. . .
**27**	Sweden	0.01483	0.00940	0.01342	0.00989	0.01897	0.01441

Table 2 presents the normalized health security scores for 27 EU countries based on the 2019 dataset (using Equation (2)). Standardization allows for comparison across different indicators by scaling the scores relative to the overall distribution.

**Table 3.** Entropy values, degrees of divergence and final weights of for HeS Indicators.

Process	N	Country	Prevention	Detection and reporting	Rapid response	Health system	Compliance with norms	Risk environment
$n_{\mathrm{ij}}$ *ln ( $n_{\mathrm{ij}})$	**1**	Austria	-0.124	-0.104	-0.112	-0.125	-0.125	-0.136
	**2**	Belgium	-0.131	-0.130	-0.127	-0.141	-0.121	-0.127
	**3**	Bulgaria	-0.145	-0.144	-0.114	-0.132	-0.132	-0.110
	**4**	Croatia	-0.121	-0.102	-0.093	-0.121	-0.113	-0.113
	**.** **.** **.**	. . .	. . .	. . .	. . .	. . .	. . .	. . .
	**27**	Sweden	-0.164	-0.149	-0.109	-0.124	-0.132	-0.133
Sum			-3.27368	-3.24666	-3.26812	-3.27420	-3.29006	-3.29021
Entj			0.99328	0.98508	0.99159	0.99344	0.99825	0.99829
1-Entj			0.007	0.015	0.008	0.007	0.002	0.002
Wj			0.168	0.372	0.210	0.164	0.044	0.043

Table 3 summarizes the entropy values and degrees of divergence for each health security indicator, along with their final weights as determined by the Entropy method (Step 3). Higher weights indicate greater importance in distinguishing health security performance among countries.

The iterative procedure of the entropy method was replicated 3 times for 2019, 2021, and the aggregated 2017–2019 timeframe.

### 2.3 Quantifying regional disparities in health security priorities in the African and Eastern mediterranean regions

To compare the entropy weighting results with previous regional research from the African region ^5^ and the Eastern Mediterranean Region (EMR), ^6^ the iterative procedure of the entropy method was replicated six additional times (for 2 regions across 3 time periods: 2019, 2021, and the aggregated timeframe of 2017–2019). Detailed calculations for this step are available in the supplementary file (Step 2 –Sheets 2.2, and 2.3). ^42^

### 2.4 Evaluating and prioritizing countries using Entropy-CoCoSo model

This study applies the Combined Compromise Solution (CoCoSo) framework to evaluate health security performance across EU. As a robust MCDM technique, the CoCoSo method ^43^ integrates the advantages of the simple additive weighting (SAW) model and the exponentially weighted product (EWP) approach. ^43^ This ranking method is further enhanced by integrating the entropy method, which objectively determines the weights and decision results using the CoCoSo method. ^36^ The entropy-CoCoSo approach has been widely applied in diverse fields, including engineering sustainability selection ^36^ and spatial-temporal analysis of business environments. ^33^ It offers enhanced precision and a finer level of differentiation between alternatives than other MCDM methods. ^43^

The CoCoSo approach comprises the following four key steps ^43^:

• Step 1: Formulating DM (G) and its normalized DM (N)

These matrices are identical to the existing decision matrices provided in (1) and (5):

nij={gij−gj−gj+−gj−,Cisabenefit criteriagij−gj+gj−−gj+,Cisadesirable criteria. (5)

Here, $g_{\mathrm{ij}}$ represents the rating of alternative (Country) i for criterion j, with $g_{j}^{+}$ and $g_{j}^{-}$ corresponding to the highest and lowest criterion-specific scores across all alternatives.

• Step 2: Computing the weighted comparability sequences as follows:
$${\mathrm{CS}}_{i}=\sum_{j=1}^{n}w_{j}n_{\mathrm{ij}},$$ (6)
$$P_{i}=\sum_{j=1}^{n}{\left(n_{\mathrm{ij}}\right)}^{w_{j}}.$$ (7)

Here, the weighting factor ( $w_{j}$ ) represents the priority (Significance **)** given to the j-th criterion, while $\left(n_{\mathrm{ij}}\right)$ captures the normalized score of the i-th alternative for the j-th evaluation criterion.

• Step 3: Determining priority weights

This phase involves deriving three distinct performance metrics through the following formulations:

$$k_{\mathrm{ia}}=\left(P_{i}+S_{i}\right)/\sum_{i=1}^{m}\left(P_{i}+S_{i}\right),$$ (8)

$$k_{\mathrm{ib}}=\left(S_{i}/\min\;S_{i}\right)+\left(P_{i}/\min\;P_{i}\right),$$ (9)

$$k_{\mathrm{ic}}=\frac{\left(\lambda \left(S_{i}\right)+\left(1-\lambda \right)P_{i}\right)}{\left(\lambda \;\left(\max\;S_{i}\right)+\left(1-\lambda \right)\max\;P_{i}\right)}.$$ (10)

Equation (A) aggregates normalized outcomes from additive and multiplicative weighting techniques. Equation (B) quantifies comparative deviations of each method’s results relative to their minima. Equation (C) harmonizes the trade-off between additive (Si) and multiplicative (Pi) dominance using a tuning coefficient α, which spans 0 to 1. This coefficient enables analysts to calibrate the dominance of either method: α = 0.5 ensures parity, while deviations skew emphasis toward one technique.

• Step 4: Generating unified composite score index and prioritization

A consolidated performance index is derived via Equation (11). Entities are then sequenced hierarchically by descending index magnitude:

$$C_{i}=\left({\left(k_{\mathrm{ia}}+k_{\mathrm{ib}}+k_{\mathrm{ic}}\right)}^{\frac{1}{3}}\right)+\left(\frac{\left(k_{\mathrm{ia}}+k_{\mathrm{ib}}+k_{\mathrm{ic}}\right)}{3}\right).$$ (11)

This research utilized a hybrid entropy-CoCoSo technique to assess and hierarchically classify nations according to their health security efficacy over three distinct timeframes (2017–2019, 2019, and 2021). The methodology evaluated national readiness and resilience in mitigating global health risks. For the 2019 EU regional analysis, a focused dataset encompassing 27 countries ( Table 1) underwent normalization via Equation 5 ( Table 4). Subsequent computational stages involved deriving weighted aggregate scores ( Equations 6– 7, Tables 5– 6) and synthesizing multi-dimensional appraisal metrics ( Equations 8– 10, Table 7). Composite indices derived from Equation 11 facilitated the hierarchical ordering of nations based on their overall performance scores. Detailed calculations for this algorithm are available in the supplementary file (Step 3-Sheet 3.1). ^42^

**Table 4.** Normalized HeS scores for EU Countries based on (2019-dataset).

N	Country	Prevention	Detection and reporting	Rapid response	Health system	Compliance with norms	Risk environment
**1**	Austria	0.45726	0.35902	0.32800	0.67955	0.45490	1.00000
**2**	Belgium	0.54076	0.62406	0.52000	0.91364	0.32549	0.74286
**3**	Bulgaria	0.72366	0.78947	0.35000	0.77727	0.67059	0.26984
**4**	Croatia	0.41750	0.34023	0.11000	0.62045	0.10588	0.35556
**.** **.** **.**	. . .	. . .	. . .	. . .	. . .	. . .	. . .
**27**	Sweden	1.00000	0.84398	0.29200	0.67045	0.67059	0.91429

Table 4 lists the normalized health security scores for EU countries, illustrating each country's performance across six indicators in 2019 (using Equation (5)).

**Table 5.** Weighted comparability sequence scores (CSi).

N	Country	Prevention	Detection and reporting	Rapid response	Health system	Compliance with norms	Risk environment	CSi
**1**	Austria	0.077	0.134	0.069	0.111	0.020	0.043	0.453
**2**	Belgium	0.091	0.232	0.109	0.150	0.014	0.032	0.628
**3**	Bulgaria	0.121	0.294	0.073	0.127	0.029	0.011	0.657
**4**	Croatia	0.070	0.127	0.023	0.102	0.005	0.015	0.341
**.** **.** **.**	. . .	. . .	. . .	. . .	. . .	. . .	. . .	. . .
**27**	Sweden	0.168	0.314	0.061	0.110	0.029	0.039	0.721

Table 5 provides the weighted comparability sequence scores for each EU country, calculated using the Entropy-CoCoSo method (Based on Equation (6)). These scores reflect the relative significance of each health security indicator in the overall ranking.

**Table 6.** Squared weighted comparability sequence scores (Pi).

N	Country	Prevention	Detection and reporting	Rapid response	Health system	Compliance with norms	Risk environment	Pi
**1**	Austria	0.877	0.683	0.791	0.939	0.966	1.000	5.256
**2**	Belgium	0.902	0.839	0.872	0.985	0.952	0.987	5.538
**3**	Bulgaria	0.947	0.916	0.802	0.960	0.983	0.946	5.553
**4**	Croatia	0.864	0.669	0.629	0.925	0.906	0.957	4.951
**.** **.** **.**	. . .	. . .	. . .	. . .	. . .	. . .	. . .	. . .
**27**	Sweden	1.000	0.939	0.772	0.937	0.983	0.996	5.627

Table 6 illustrates the squared weighted comparability scores for EU countries (Based on Equation (7)).These scores indicate the performance of each country across the health security indicators, emphasizing areas of strength and weakness.

**Table 7.** Final composite scores (ci) and ranks (Ri) of countries.

N	Country	$k_{\mathrm{ia}}$	$k_{\mathrm{ib}}$	$k_{\mathrm{ic}}$	Ci	Ri
**1**	Austria	0.038	15.125	0.851	6.123	17
**2**	Belgium	0.041	20.247	0.919	7.979	10
**3**	Bulgaria	0.041	21.090	0.926	8.280	8
**4**	Croatia	0.035	11.798	0.789	4.894	23
**.** **.** **.**	. . .	. . .	. . .	. . .	. . .	. . .
**27**	Sweden	0.042	22.966	0.946	8.953	6

Table 7 presents the final composite scores and rankings for EU countries based on the Entropy-CoCoSo method ( Equations (8-11)).

### 2.5 K-Means clustering for EU health security rankings

K-means clustering, a sophisticated method for partitioning datasets into distinct groups, ^28^ was employed to classify countries in Africa, ^5^ Western Asia, ^10^ and the Eastern Mediterranean Region ^6^ based on HeS scores of countries. This technique assigns alternatives to clusters based on similarity, minimizes within-cluster variance, and ensures that alternatives within a group exhibit comparable HeS characteristics. K-means is particularly effective for large datasets and yields clear and interpretable results, making it ideal for this analysis. ^29^

Given a composite scores vector $X=\{X_{1}$ , $\;X_{2},\ldots .,X_{m}\}$ , where each $X_{i}$ ∈R represents a data point (composite score) for an EU country under study, and m is the total number of such scores, the K-means clustering algorithm aims to partition these data points into k clusters denoted as $L=\{L_{1}$ , $\;L_{2},\ldots ..,L_{k}\}$ .

The K-means clustering methodology unfolds through sequential phases ^44, 45^:

**Step 1**: Initialize k cluster centroids ( ${\mu_{i}}^{\left(0\right)},{\mu_{i}}^{\left(0\right)},\ldots .,{\mu_{k}}^{\left(0\right)}$ ) are randomly selected from the dataset. Here, each $\mu_{k}$ represents the centroid (i.e., the mean) of cluster $\;L_{k}$ .

**Step 2**: Assign each data point $X_{i}\;$ to the cluster $\;L_{k}$ with the nearest centroid. This assignment is based on the squared Euclidean distance:

$$L_{k}^{\left(t\right)}=\{X_{i}:{\left\|X_{i}-\mu_{k}^{\left(t\right)}\right\|}^{2}\leq {\left\|X_{i}-\mu_{j}^{\left(t\right)}\right\|}^{2},\forall j=\left\{1,2,\ldots ,K\right\}\;$$ (12)

**Step 3:** Recalculate the centroid of each cluster as the mean of all points assigned to it:

$$\mu_{k}^{\left(t+1\right)}=\frac{1}{\left|L_{k}^{\left(t\right)}\right|}\sum_{x_{i}\epsilon L_{k}^{\left(t\right)}}X_{i}\;$$ (13)

**Step 4:** Repeat Steps 2 and 3 iteratively until convergence. Convergence is typically defined by one of the following criteria: (1) cluster assignments do not change between iterations, (2) centroids move less than a predefined threshold, or (3) a maximum number of iterations is reached. At each iteration, the algorithm seeks to minimize the intra-cluster variance, denoted by the objective function J, which encourages the formation of compact and well-separated clusters:

$$J=\text{argmin}\sum_{i=1,\left(x_{i}\epsilon L_{k}\right)}^{k}\sum {\left\|X_{i}-\mu_{k}\right\|}^{2}\;$$ (14)

Here, ${\left\|X_{i}-\mu_{k}\right\|}^{2}\;$ is the squared Euclidean distance between a point $X_{i}$ and the centroid of its assigned cluster $\mu_{k}$ .

In this study, we employed the Elbow Method to determine the optimal number of clusters, using the Within-Cluster Sum of Squares (WCSS) as the evaluation criterion. The “elbow” point on the WCSS curve represents the point at which the rate of decrease significantly changes, indicating diminishing returns from adding additional clusters.

**Figure 1.**
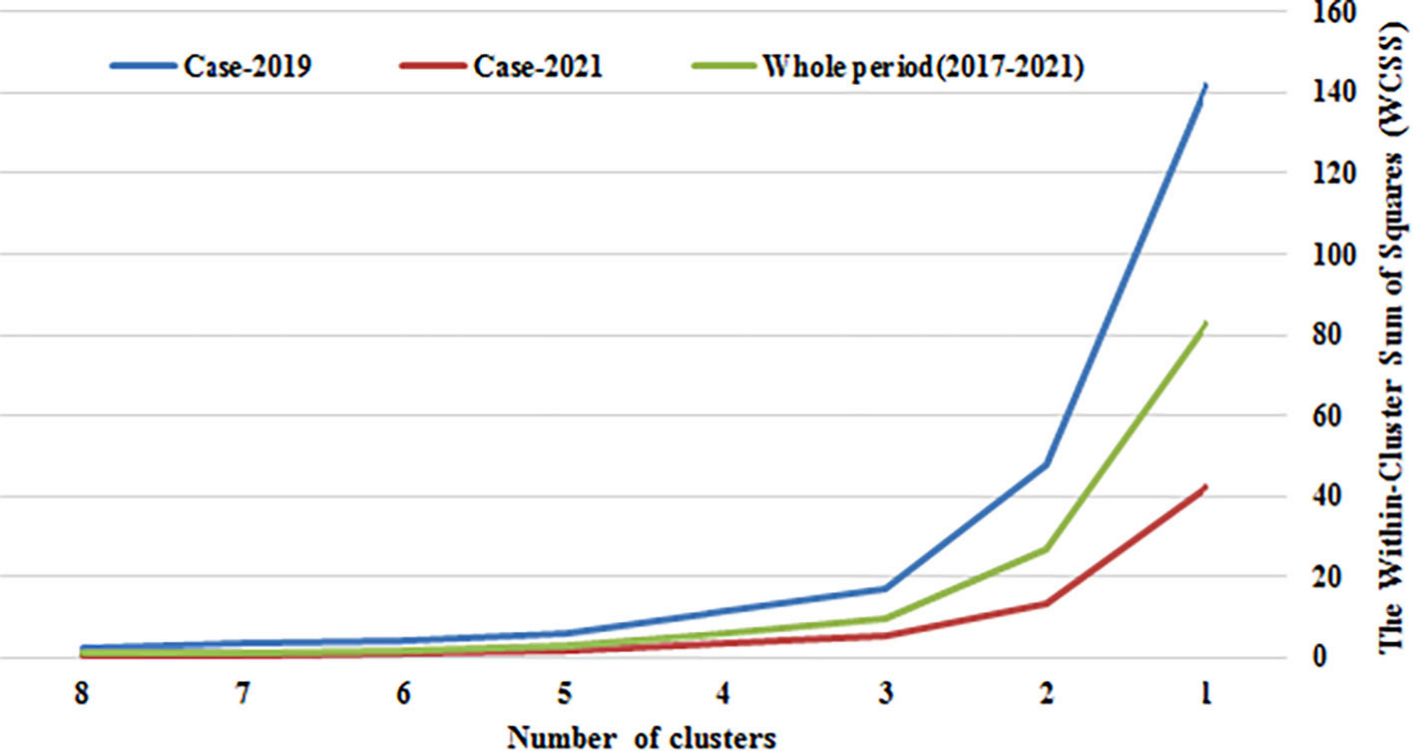
Elbow Method results for determining the optimal number of clusters. The plot displays the relationship between the number of clusters (k) and the within-cluster sum of squares (WCSS).

As illustrated in Figure 1, there was a substantial reduction in WCSS when increasing from one to two clusters, followed by a smaller yet noticeable decrease from two to three clusters. The decrease from three to four clusters was even less pronounced, with subsequent reductions continuing at a gradual pace. Consequently, the elbow point was identified between two and three clusters. Based on this analysis, we selected three clusters as the optimal number for our study, achieving a balance between minimized WCSS and diminishing marginal improvement.

In terms of performance levels, Cluster 1 represented ‘High’ performance, Cluster 2 ‘Intermediate’, and Cluster 3 ‘Dangerous’, enabling a nuanced evaluation of each country's regional standing.

The clustering method was applied 3 times to cluster EU countries based on the results of the ranking method across the three datasets. Detailed calculations for this step are available in the supplementary file (sheets: 4.1, 4.2). ^42^

## 3. Results

### 3.1 Entropy-Driven assessment of HeS priorities

**Figure 2.**
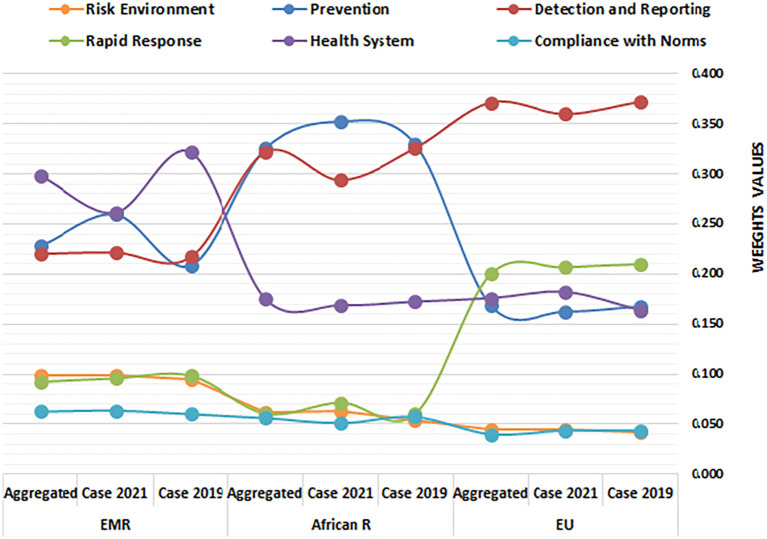
Regional prioritization of HeS dimensions: EU vs. EMR vs. African Region. This figure illustrates the entropy-weighted prioritization of health security dimensions across EU countries, the Eastern Mediterranean Region (EMR), and the African Region (2017–2021). The figure compares the relative weights of six Global Health Security Index (GHSI) indicators—Prevention, Detection & Reporting, Rapid Response, Health System, Compliance with Norms, and Risk Environment—across regions and timeframes.

The calculated entropy weights of HeS indicators for EU countries, alongside comparative weights for the African Region and Eastern Mediterranean Region (EMR), are presented in Table 8. Table 8 and Figure 2 offer critical insights into the prioritization of HeS indicators across regions. Figure 2 illustrates the weight distribution of HeS-related dimensions, comparing the EU, African Region, and EMR over three timeframes: 2019, 2021, and the aggregated period (2017–2021). Additionally, Table 9 provides tabular representations of dynamic prioritization trends, highlighting changes in indicator weights between 2019 and 2021.

**Table 8.** Regional prioritization of HeS dimensions: EU vs. EMR vs. African Region.

Data	Region	Prevention	Detection and reporting	Rapid response	Health system	Compliance with norms	Risk environment
**2019**	European Union	0.168	0.372	0.210	0.164	0.044	0.043
**2021**		0.162	0.360	0.207	0.182	0.044	0.045
**Aggregated**		0.168	0.371	0.200	0.176	0.040	0.044
**2019**	African Region	0.330	0.326	0.060	0.172	0.057	0.055
**2021**		0.352	0.294	0.072	0.169	0.051	0.063
**Aggregated**		0.325	0.322	0.061	0.175	0.056	0.061
**2019**	Eastern Mediterranean	0.209	0.217	0.098	0.322	0.060	0.095
**2021**		0.259	0.221	0.096	0.261	0.063	0.099
**Aggregated**		0.228	0.220	0.092	0.298	0.063	0.099

Table 8 presents the entropy-weighted prioritization of health security dimensions across regions. It includes weights for six GHSI indicators in the EU, Africa, and the EMR for the years 2019, 2021, and the aggregated period of 2017–2021. The table compares the relative weights of six Global Health Security Index (GHSI) indicators—Prevention, Detection & Reporting, Rapid Response, Health System, Compliance with Norms, and Risk Environment—across different regions and timeframes **.**

**Table 9.** Temporal shifts in HeS indicator weights: EU, African Region, and EMR (2019 vs. 2021).

Region	Prevention	Detection and reporting	Rapid response	Health system	Compliance with norms	Risk environment
** European Union**	-0.005	-0.013	-0.003	0.018	0.000	0.003
**African R**	0.023	-0.032	0.011	-0.004	-0.006	0.008
**Eastern Mediterranean**	0.051	0.004	-0.003	-0.060	0.004	0.004

Positive/negative values indicate increased/decreased prioritization of indicators over time.

### 3.2 EU health security rankings and cluster-based interventions

**Figure 3.**
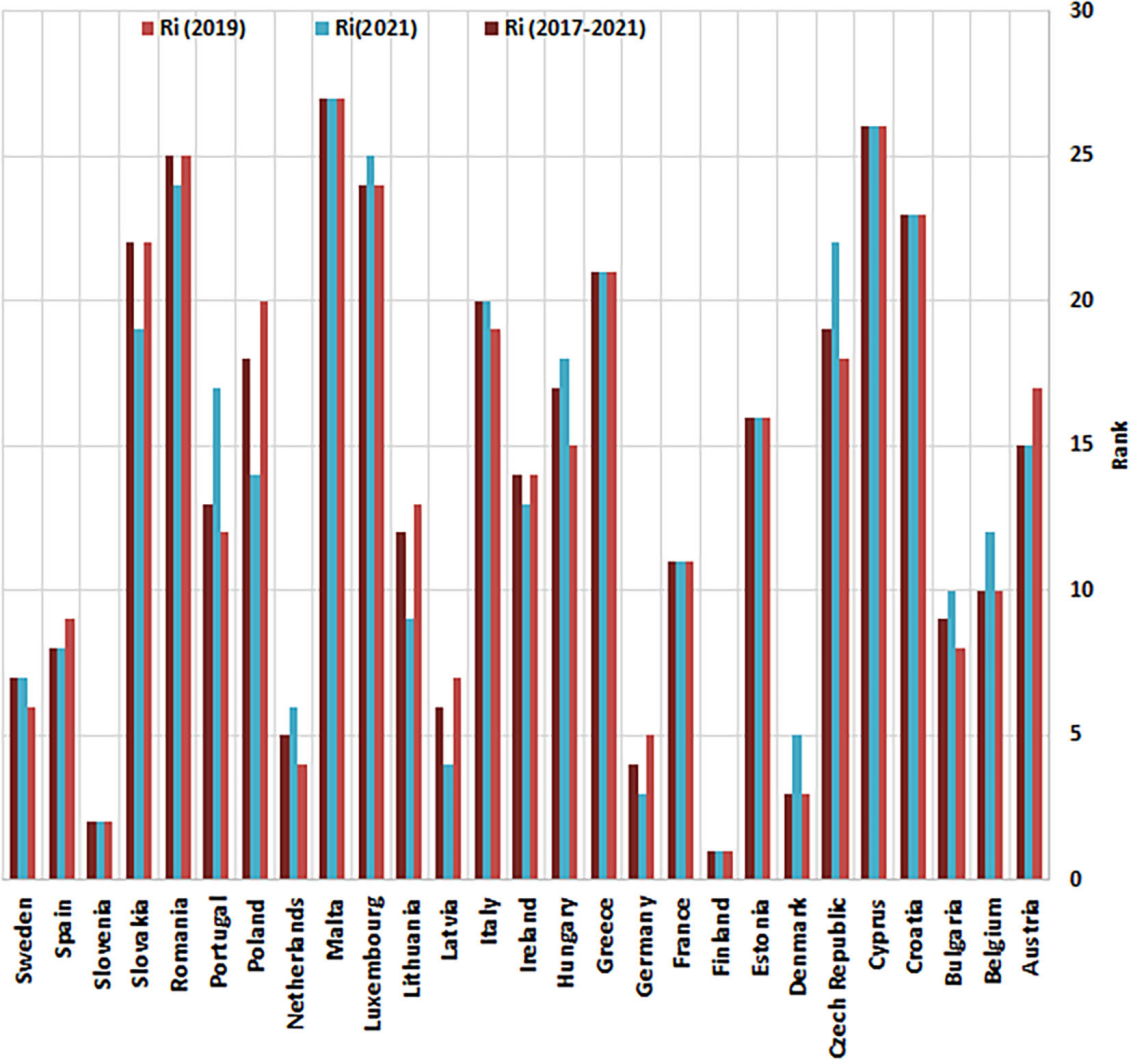
Entropy-CoCoSo-based ranking results and ranking shifts (2019–2021). This bar chart illustrates the Health Security Preparedness Rankings of non-EU European countries, encompassing the years 2019, 2021, and the entire period of 2017-2021. Countries are ranked based on their Composite CoCoSo scores, with higher values indicating superior performance. It also reflects temporal shifts in health security capabilities.

**Figure 4.**
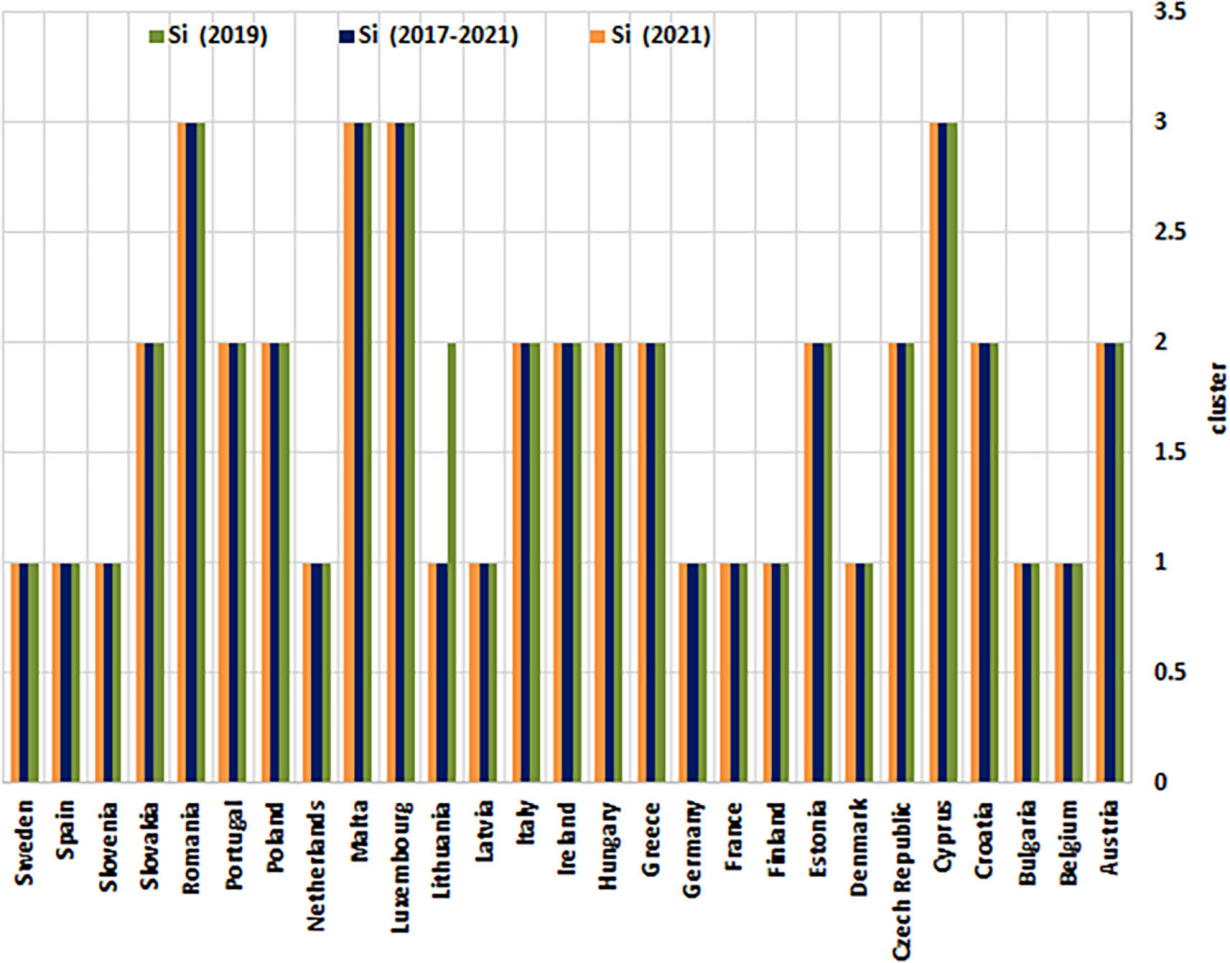
Health security performance clustering results of EU countries. This figure illustrates the K-Means Clustering results of EU countries, encompassing the years 2019, 2021, and the entire period of 2017-2021. Countries are grouped into three clusters based on their health security profiles, with Cluster 1 representing exemplary performance and Cluster 3 representing critical performance. The figure also reveals patterns of similarity and dissimilarity among countries in terms of their health security capabilities.

The HeS performance assessments (Ci) and corresponding rankings (Ri) for EU countries, derived from the Entropy-CoCoSo method, are displayed in Table 10. These evaluations cover three time frames: 2019, 2021, and the combined period of 2017–2021. Each nation is assigned to one of three performance categories (Si), ranging from S1 (indicating superior performance) to S3 (signifying a critical situation ‘Dangerous’). These tiers reflect varying degrees of outcomes, from optimal to severely inadequate. The table also highlights changes in ranking positions and tier classifications between 2019 and 2021, revealing trends of progress or regression among these nations. A visual representation of the ranking trajectories is provided in Figure 3, while Figure 4 showcases the distribution of cluster tiers across the examined states.

**Table 10.** Entropy-CoCoSo HeS Scores (C _i_), Rankings (R _i_), and Cluster Assignments (S _i_) for EU Countries.

N	State	Case 1-2019			Case 2-2021			Case 3-The whole period (2017-2021)			Shifts	
		Ci	Ri	Si	Ci	Ri	Si	Ci	Ri	Si	Ri	Si
A-1	Austria	6.123	17	2	3.910	15	2	4.969	15	2	2	0
A-2	Belgium	7.979	10	1	4.637	12	1	6.210	10	1	-2	0
A-3	Bulgaria	8.280	8	1	4.886	10	1	6.502	9	1	-2	0
A-4	Croatia	4.894	23	2	3.002	23	2	3.881	23	2	0	0
A-5	Cyprus	2.280	26	3	1.864	26	3	1.933	26	3	0	0
A-6	Czech Republic	5.963	18	2	3.419	22	2	4.711	19	2	-4	0
A-7	Denmark	9.581	3	1	5.489	5	1	7.426	3	1	-2	0
A-8	Estonia	6.157	16	2	3.890	16	2	4.932	16	2	0	0
A-9	Finland	10.442	1	1	6.275	1	1	8.262	1	1	0	0
A-10	France	7.745	11	1	4.740	11	1	6.189	11	1	0	0
A-11	Germany	9.250	5	1	5.571	3	1	7.319	4	1	2	0
A-12	Greece	5.617	21	2	3.473	21	2	4.483	21	2	0	0
A-13	Hungary	6.169	15	2	3.776	18	2	4.887	17	2	-3	0
A-14	Ireland	6.299	14	2	4.037	13	2	5.129	14	2	1	0
A-15	Italy	5.841	19	2	3.622	20	2	4.644	20	2	-1	0
A-16	Latvia	8.598	7	1	5.502	4	1	7.042	6	1	3	0
A-17	Lithuania	6.912	13	2	4.944	9	1	5.969	12	1	4	1
A-18	Luxembourg	3.935	24	3	2.523	25	3	3.148	24	3	-1	0
A-19	Malta	1.025	27	3	1.109	27	3	1.085	27	3	0	0
A-20	Netherlands	9.516	4	1	5.360	6	1	7.289	5	1	-2	0
A-21	Poland	5.633	20	2	3.951	14	2	4.795	18	2	6	0
A-22	Portugal	6.984	12	2	3.801	17	2	5.231	13	2	-5	0
A-23	Romania	3.736	25	3	2.587	24	3	3.117	25	3	1	0
A-24	Slovakia	5.083	22	2	3.767	19	2	4.473	22	2	3	0
A-25	Slovenia	9.853	2	1	6.140	2	1	7.965	2	1	0	0
A-26	Spain	8.116	9	1	5.164	8	1	6.603	8	1	1	0
A-27	Sweden	8.953	6	1	5.236	7	1	6.999	7	1	-1	0

Table 10 illustrates the results of the ranking and clustering analysis of health security performance in EU countries, encompassing the years 2019, 2021, and the entire period of 2017-2021. The analysis yields a set of key metrics, including the final Entropy-CoCoSo scores (Ci), ranks (Ri), and cluster assignments (Si). Notably, higher Ci and Ri values signify superior performance, whereas cluster membership is categorized into five tiers, ranging from Cluster 1 (exemplary performance) to Cluster 3 (critical performance).

### 3.3 Comparison of Entropy-CoCoSo with other Entropy-based MCDM methods

This study evaluated the efficacy of the Entropy-CoCoSo (E-CoCoSo) method in comparison to four other entropy-based methods—Entropy-TOPSIS, Entropy-EDAS, Entropy-WASPAS, and Entropy-VIKOR—across three distinct periods (2019, 2021, and 2017–2021). Figure 5.a, 5.b, and 5.c depict the rankings of the 27 EU countries as alternatives for each scenario, while Table 11 presents the corresponding Spearman’s rank correlation coefficients to validate the consistency of the methods.

**Figure 5.a.**
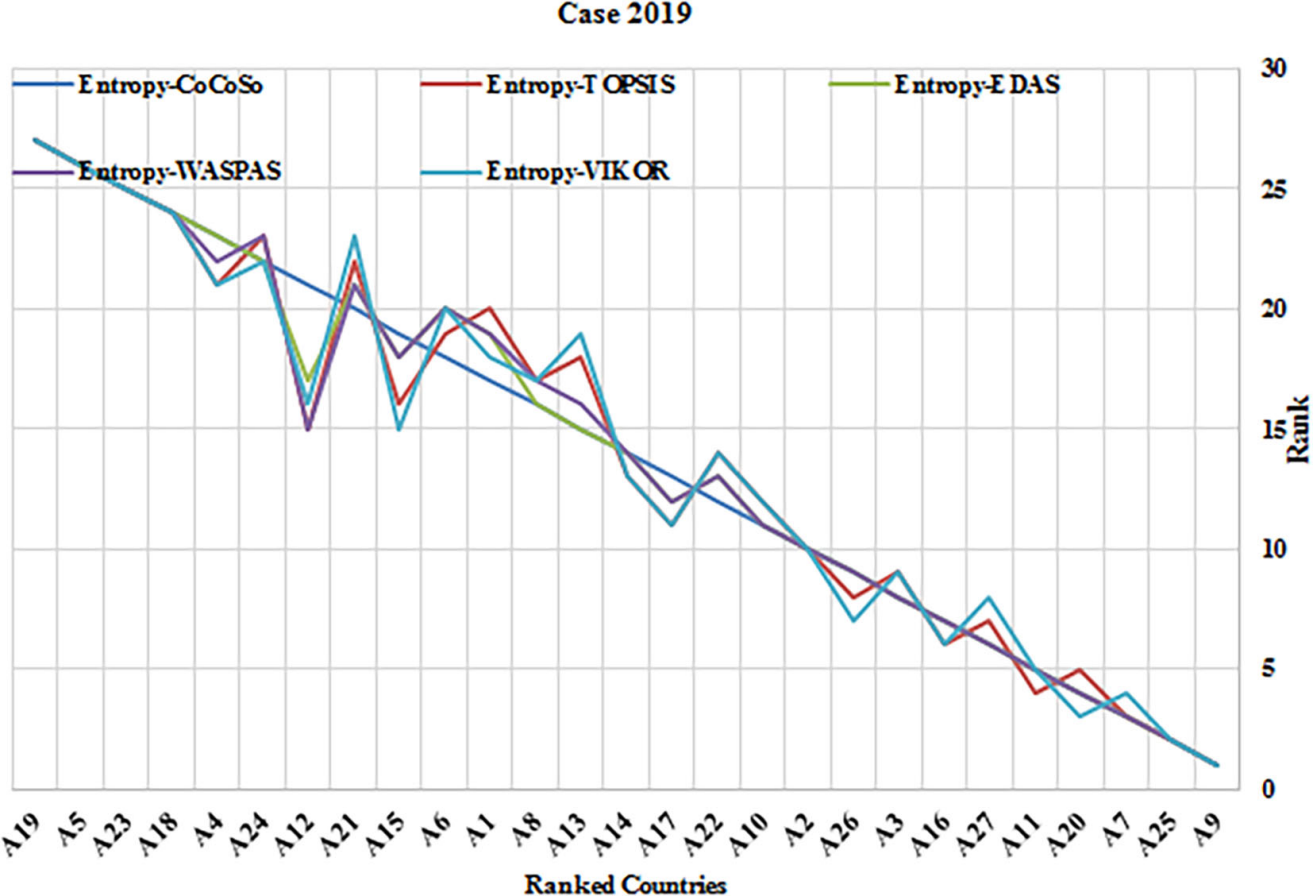
Rankings of 27 EU countries using Entropy-CoCoSo, TOPSIS, EDAS, WASPAS, and VIKOR methods for the year 2019. This Chart demonstrate consistency across different multi-criteria decision-making approaches in evaluating health security performance. It compares the EU country rankings using Entropy-CoCoSo, Entropy-TOPSIS, Entropy-EDAS, Entropy-WASPAS, and Entropy-VIKOR methods for 2019.

**Figure 5.b.**
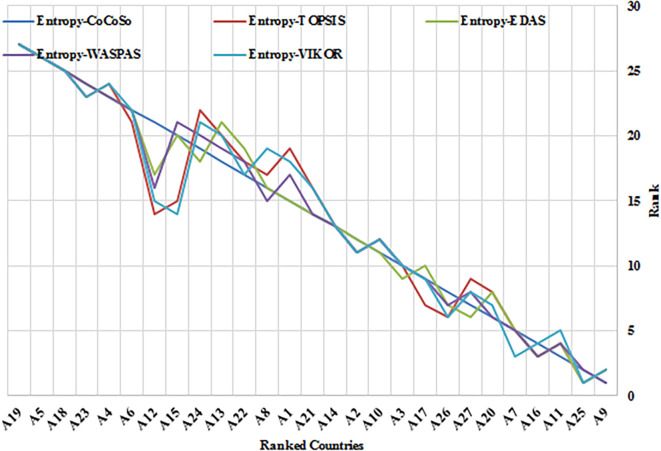
Rankings of 27 EU countries using Entropy-CoCoSo, TOPSIS, EDAS, WASPAS, and VIKOR methods for the year 2021. This Chart demonstrate consistency across different multi-criteria decision-making approaches in evaluating health security performance. It compares the EU country rankings using Entropy-CoCoSo, Entropy-TOPSIS, Entropy-EDAS, Entropy-WASPAS, and Entropy-VIKOR methods for 2021.

**Figure 5.c.**
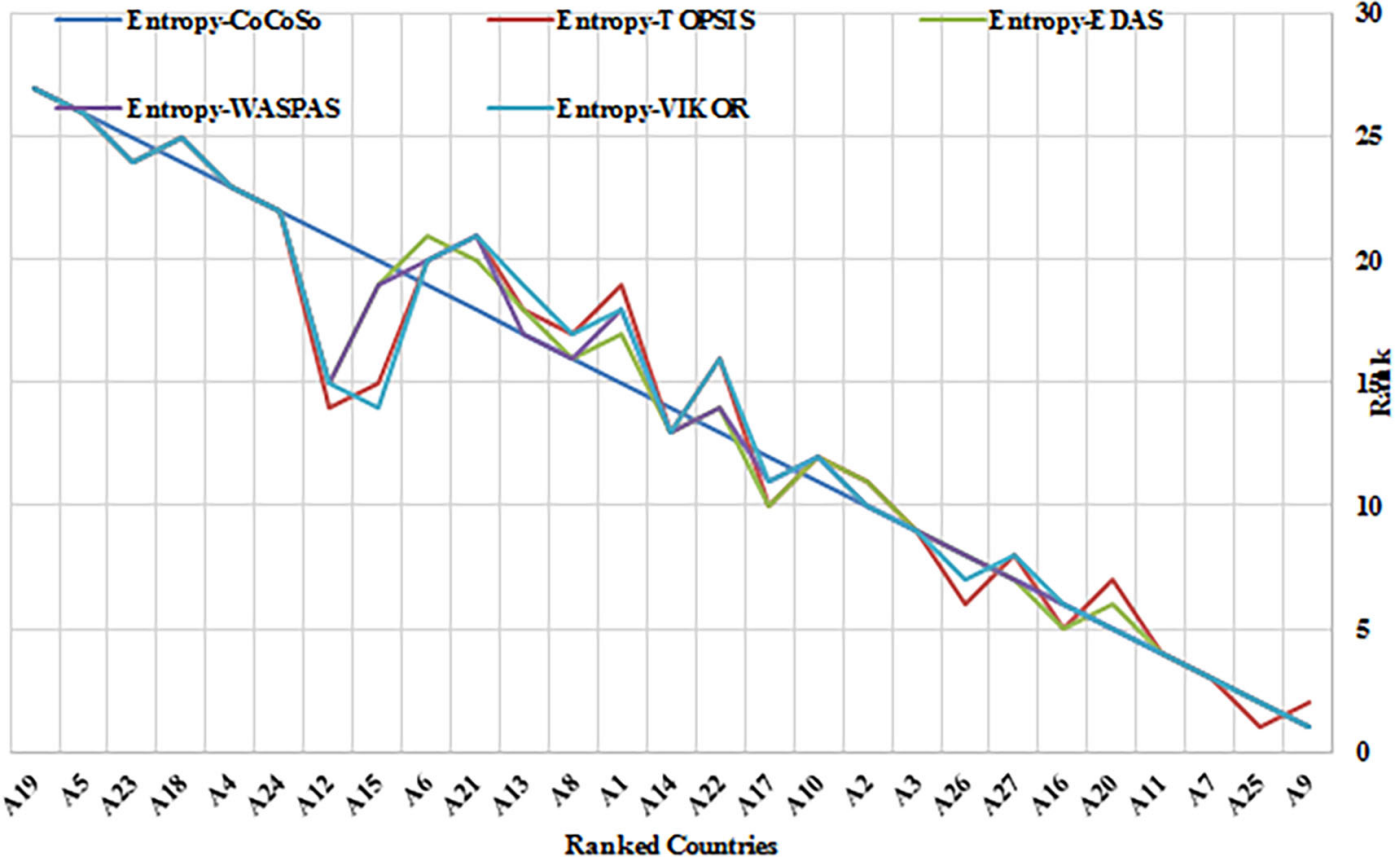
Rankings of 27 EU countries using Entropy-CoCoSo, TOPSIS, EDAS, WASPAS, and VIKOR methods for the period 2017-2021. This chart reflects the consistency among different multi-criteria decision-making frameworks in assessing health security performance. It provides a comparative analysis of EU country rankings using the Entropy-CoCoSo, Entropy-TOPSIS, Entropy-EDAS, Entropy-WASPAS, and Entropy-VIKOR methods over the specified period. Note: A1 – Austria, A2 – Belgium, A3 – Bulgaria, A4 – Croatia, A5 – Cyprus, A6 – Czech Republic, A7 – Denmark, A8 – Estonia, A9 – Finland, A10 – France, A11 – Germany, A12 – Greece, A13 – Hungary, A14 – Ireland, A15 – Italy, A16 – Latvia, A17 – Lithuania, A18 – Luxembourg, A19 – Malta, A20 – Netherlands, A21 – Poland, A22 – Portugal, A23 – Romania, A24 – Slovakia, A25 – Slovenia, A26 – Spain, and A27 – Sweden.

**Table 11.** Spearman’s ranking coefficients of correlation (Average).

Spearman’s rho	Entropy-CoCoSo	Entropy-TOPSIS	Entropy-EDAS	Entropy WASPAS	Entropy-VIKOR
Entropy-CoCoSo	1	0.963777	0.9866	0.9843305	0.96622
Entropy -TOPSIS	0.963777	1	0.9827	0.986569	0.99451
Entropy –EDAS	0.986569	0.982702	1	0.9953195	0.98107
Entropy WASPAS	0.98433	0.986569	0.9953	1	0.98331
Entropy -VIKOR	0.966219	0.994505	0.9811	0.983313	1

Table 11 presents the average Spearman’s rank correlation coefficients between the Entropy-CoCoSo method and four other entropy-based multi-criteria decision-making methods: Entropy-TOPSIS, Entropy-EDAS, Entropy-WASPAS, and Entropy-VIKOR. The average coefficients indicate the degree of consistency in rankings across the different methods for the years 2019, 2021, and the aggregated period of 2017–2021. Higher values signify stronger agreement between the methods.

## 4. Discussion

### 4.1 Comparative weighting of health security domains: EU, Africa, and EMR perspectives

The model's first step establishes a hierarchy of importance for health security indicators, aiding decision-makers in resource allocation and prioritization. Weighted indicators enable thorough analysis of complex systems, considering each indicator's impact on outcomes. Longitudinal analysis across 2019, 2021, and 2017-2021 provides insights into evolving health security priorities, helping identify trends, shifts, and emerging challenges. Regional comparisons (EU, Africa, and EMR) highlight disparities in priorities and resource allocation, informing targeted global health initiatives. These evaluations promote international collaboration and knowledge sharing, with successful regions serving as models. Recognizing regional differences allows for tailored, context-specific approaches to health security. Dynamic analysis of indicator weights between 2019 and 2021, compared across regions and sub-regions, offers a comprehensive understanding of evolving health security priorities. Policymakers can use these insights to identify trends, assess intervention effectiveness, and design responsive strategies.

***4.1.1 The entropy-derived weights for EU countries***

The entropy-derived weights reveal that Detection & Reporting (0.36–0.37) and Rapid Response (0.20–0.21) are the most influential criteria in differentiating health security performance across EU countries, indicating significant disparities in surveillance capabilities and emergency responsiveness between states. The slight decline in Detection & Reporting’s weight by 2021 (0.372 to 0.360) suggests reduced variability in this domain, potentially reflecting pandemic-driven convergence in surveillance systems. This convergence may be attributed to the EU's efforts to harmonize data collection and reporting through initiatives like the European Surveillance System (TESSy).

In contrast, the rising weight of health systems (0.164 to 0.182) signals growing divergence in healthcare infrastructure resilience post-2019, likely exacerbated by uneven pandemic recovery efforts. This divergence is concerning, as it suggests that some member states are falling behind in their ability to provide essential healthcare services during emergencies, potentially due to underinvestment in healthcare infrastructure and shortages of healthcare professionals. ^46^ Policymakers should prioritize investments in strengthening healthcare systems in the most vulnerable member states to ensure equitable access to care during future health crises. ^47^

Prevention’s stable weight (~0.16) implies moderate but consistent differentiation, while compliance with norms and risk environments—persistently low weights (~0.04)—highlights their negligible role in distinguishing performance, either due to uniform underperformance or insufficient data variability.

These weights prioritize addressing disparities in high-impact areas (detection & reporting, rapid response) over uniformly improving low-weight domains. Policymakers should thus focus on harmonizing detection capacities and emergency coordination mechanisms to reduce inter-country gaps while investigating systemic weaknesses in compliance and risk environments that the model currently overlooks. The aggregated weights’ stability underscores the enduring centrality of detection & reporting as a benchmark, urging targeted investments to sustain its discriminative power in EU health security evaluations.

***4.1.2 Comparative analysis of EU and African health security priorities***

The entropy weights highlight stark contrasts in the factors driving health security differentiation between the EU and Africa. In Africa, prevention (0.325–0.352) and detection & reporting (0.294–0.326) dominate as the most influential criteria, reflecting significant disparities in preemptive measures and surveillance capabilities across African nations. ^5^ By contrast, the EU’s model prioritizes Detection & Reporting (0.360–0.372) and Rapid Response (0.20–0.21), underscoring variability in crisis coordination and real-time surveillance as key differentiators. Notably, Rapid Response is far less impactful in Africa (0.060–0.072 vs. the EU’s ~0.20), suggesting emergency mobilization systems are either underdeveloped or uniformly weak across the continent. Temporal trends also diverge. Africa’s prevention weight increased by 2021 (0.330 → 0.352), likely driven by pandemic-era investments in preventive care, while its detection weight declined (0.326 → 0.294), signaling reduced variability in surveillance systems. Conversely, the EU saw rising health system weights (0.164 → 0.182), emphasizing post-2019 disparities in healthcare resilience but stagnant prevention influence (~0.16). Both regions show minimal differentiation from compliance with norms and risk environments (weights ~0.04–0.06), though Africa’s slightly higher values hint at marginally greater variability in regulatory adherence or environmental risks.

The contrasting health security priorities between the EU and Africa have important implications for potential collaboration. The EU could provide technical assistance and financial support to help African countries strengthen their surveillance systems and improve their capacity for rapid response. At the same time, African countries could share their expertise in managing infectious diseases and implementing community-based prevention programs. By working together, the EU and Africa can enhance global health security.

***4.1.3 Comparative analysis with the Eastern Mediterranean Region (EMR)***

The EMR’s entropy weights reveal Health System (0.261–0.322) as the dominant driver of health security differentiation, contrasting sharply with the EU’s focus on Detection & Reporting and Africa’s emphasis on Prevention. While EMR’s health system weight declined post-2019 (0.322 → 0.261), due to pandemic strain on infrastructure, ^6^ prevention gained prominence (0.209 → 0.259), reflecting growing disparities in preemptive measures like vaccine equity or sanitation access. EMR’s Detection & Reporting weights (0.217–0.221) are lower than the EU’s (0.36–0.37) but higher than Africa’s (0.29–0.33), suggesting moderate variability in surveillance systems. Rapid Response (0.092–0.098) remains a minor differentiator, similar to Africa but far below the EU, indicating widespread emergency coordination gaps. Compliance with Norms and Risk Environment (~0.06–0.10) holds marginally higher weights than in the EU or Africa, hinting at slightly greater variability in regulatory adherence or climate-linked health risks.

***4.1.4 EU Health security priorities: Evolution and regional divergence in a post-pandemic context (2019–2021)***

The EU’s dynamic prioritization of health security indicators between 2019 and 2021 reveals a nuanced evolution shaped by the COVID-19 pandemic. Detection and Reporting (-0.013) and Prevention (-0.005) saw modest declines in weight, potentially reflecting a pandemic-induced homogenization of surveillance and preventive measures across member states as they adopted standardized responses. However, this apparent convergence should be interpreted cautiously, as it may mask underlying disparities in the effectiveness of these measures. In contrast, Health System (+0.018) gained importance, signaling growing post-2019 disparities in healthcare resilience, likely exacerbated by uneven national recovery efforts and pre-existing vulnerabilities in certain member states. ^46^

Compared to Africa, where prevention surged (+0.023) and detection plummeted (-0.032), the EU’s prioritization diverges sharply. This divergence underscores the contrasting realities and strategic choices facing the two regions: Africa's focus shifted toward preemptive measures, likely driven by the need to compensate for fragmented and under-resourced surveillance systems, ^48, 49^ while the EU, with its stronger baseline detection capabilities, experienced a relative decline in the *differentiating power* of those systems.

The Eastern Mediterranean Region (EMR) diverges further, with Prevention (+0.051) rising steeply and Health System (-0.060) collapsing as a differentiator. This dramatic shift likely reflects the severe strain placed on healthcare infrastructure in the EMR by the pandemic, coupled with a strategic emphasis on prevention in resource-constrained settings. Unlike the EU’s stable compliance, both Africa (-0.006) and EMR (+0.004) show minimal regulatory prioritization, highlighting the EU’s relative institutional maturity, even though all regions share persistent gaps in Rapid Response (-0.003 to -0.007). This shared weakness in rapid response capabilities underscores the urgent need for improved cross-border coordination and resource mobilization to effectively address future health emergencies. ^5, 6^

### 4.2 Discussion of HeS performance ranking results

The Health Security Index (HSI) rankings for EU countries revealed significant variability between 2019 and 2021. Nine EU nations (33.33%)—including Austria, Germany, Ireland, Latvia, Lithuania, Poland, Romania, Slovakia, and Spain—exhibited improved rank values, reflecting tangible progress in specific health security measures or a more effective overall pandemic response. Conversely, the rankings remained unchanged in eight countries (29%), such as Croatia, Cyprus, Estonia, Finland, France, Greece, Malta, and Slovenia, suggesting either a stable baseline performance or a balancing of gains and losses across different HSI indicators. However, a decline was observed in ten EU countries (37%), including Belgium, Bulgaria, Czech Republic, Denmark, Hungary, Italy, Luxembourg, Netherlands, Portugal, and Sweden, signaling potential vulnerabilities that were exposed or exacerbated during the pandemic and warrant further investigation to identify specific areas for improvement in their health security systems ( Figure 3). These shifts in rankings underscore the dynamic nature of health security and the importance of continuous monitoring and adaptation. Poland's significant improvement, surging six ranks (20→14), demonstrates the potential for rapid progress through targeted investments and policy reforms, such as increased funding for detection and reporting infrastructure or improved health system capabilities. ^9^

The advancements made by Lithuania (+4) and Latvia (+3), along with moderate gains in Central Europe (Slovakia +3, Austria/Germany +2) and Western nations (Ireland/Spain +1), underscore the diverse approaches to health security within the EU, reflecting varying national priorities, resource allocations, and pre-existing strengths and weaknesses.

While Finland and Slovenia maintained their leading positions in EU health security rankings from 2019 to 2021, suggesting robust and resilient health security systems, other countries experienced notable shifts that highlight the need for vigilance. Denmark's fall from 3rd to 5th place, potentially due to weakened prevention and response measures as resources were diverted to managing the acute phase of the pandemic, illustrates the importance of maintaining robust health security systems across all domains, even during times of crisis. ^9^ Similarly, the Netherlands' critical declines in various areas, including trade and travel restrictions (scores: 100→0), likely reflecting a shift in policy toward less restrictive measures, and zoonotic management, expose potential vulnerabilities in surveillance and mitigation strategies that require careful re-evaluation.

The consistently low rankings of Luxembourg, Romania, Cyprus, and Malta reveal systemic challenges in their health security frameworks. These countries scored poorly in critical areas such as biosecurity, laboratory systems, and emergency financing, highlighting the need for comprehensive reforms. However, each of these nations also demonstrated niche strengths, such as Romania's zoonotic surveillance and Malta's workforce strategy, which could serve as foundations for improvement.

The wide variation in scores within countries reflects uneven priorities or resource allocation in health security measures, rather than uniform strengths or weaknesses. While shared strengths in urbanization and road networks may facilitate rapid response in some areas, persistent gaps in biosecurity, laboratory capacity, and emergency preparedness require targeted interventions and tailored strategies to address specific national needs. Smaller nations like Luxembourg and Malta face unique resource constraints, necessitating innovative approaches and potential collaboration with larger member states to enhance their health security capabilities.

Addressing these deficits while leveraging existing strengths is crucial for improving resilience against future health crises across the EU. The diverse performance of EU countries in the Health Security Index underscores the need for continued collaboration, knowledge sharing of best practices, and targeted improvements to enhance overall health security within the region. This includes strengthening cross-border coordination, investing in research and development, and promoting a culture of preparedness at all levels of society.

### 4.3 Comparison of E-CoCoSo with other entropy-based MCDM methods

Overall, the E-CoCoSo method exhibited excellent concordance with the other approaches ( Figure 5.a, 5.b, and 5.c), particularly with E-EDAS (ρ = 0.9866) and E-WASPAS (ρ = 0.9843) ( Table 11). The correlation with E-TOPSIS was also notably strong (ρ = 0.9638), whereas the lowest—yet still high—correlation was observed with E-VIKOR (ρ = 0.9662).

A detailed comparison of E-CoCoSo and E-EDAS across the three cases revealed a remarkable level of consistency and similarity. The rankings produced by E-CoCoSo closely mirrored those of E-EDAS, especially among the top-performing alternatives such as A9, A25, A7, and A11. Minor differences appeared mainly among the mid- and lower-ranked alternatives; however, these variations were minimal and did not substantially impact the overall ranking trends. This high level of alignment indicates that E-CoCoSo provides a comparable degree of reliability and robustness to the well-established E-EDAS method across different evaluation periods.

Similarly, the comparison between E-CoCoSo and Entropy-WASPAS confirms a very high degree of agreement, as reflected by a Spearman’s correlation coefficient of ρ = 0.9843. Both methods consistently ranked the leading alternatives across all three cases, with particularly close alignment in the first and third cases. Although minor differences were again noted among the lower-ranked alternatives, the overall ranking structure remained stable, reaffirming the strong consistency of the E-CoCoSo method relative to Entropy-WASPAS.

Although the correlation between E-CoCoSo and E-VIKOR was slightly lower, it remained strong, suggesting that while both methods generally agree, some differences in ranking outcomes exist. These discrepancies can be attributed to variations in the underlying algorithmic structures and aggregation techniques employed by each method, highlighting that each approach retains unique characteristics.

Furthermore, among all method pairs, the strongest overall correlation was observed between Entropy-EDAS and Entropy-WASPAS (ρ = 0.9953), underscoring their extremely similar outputs. Entropy-TOPSIS and Entropy-VIKOR also exhibited a near-perfect correlation (ρ = 0.9945), emphasizing their strong mutual consistency. Taken together, the consistently high Spearman’s coefficients across all comparisons confirm that entropy-based MCDM methods, including E-CoCoSo, produce highly comparable and reliable decision-making outcomes, thus reinforcing the validity of E-CoCoSo as a robust tool for multi-criteria evaluations.

### 4.4 Health security performance: Insights and patterns across three clusters

**Figure 6.**
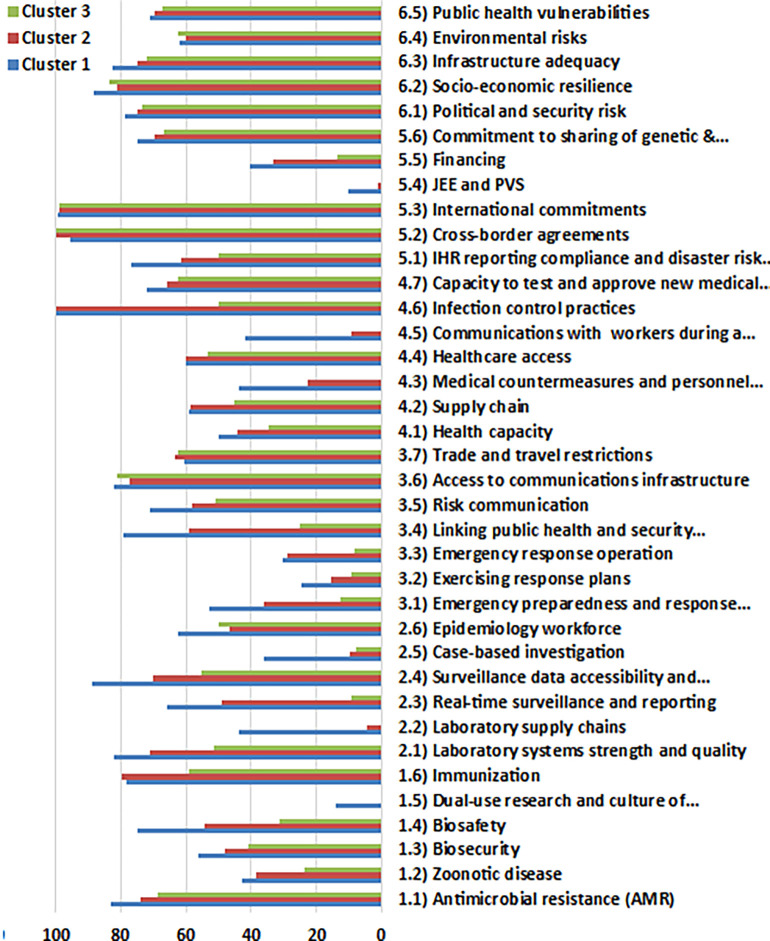
Average health security performance scores across the clusters. This figure compares the mean scores of six HeS sub-indicators for each cluster (High, Intermediate, and Dangerous). The bar chart contrasts the High, Intermediate, and Dangerous tiers across multiple indicators, highlighting strengths and weaknesses in regional preparedness. Scores range from 0 to 100, with higher values indicating stronger performance. The visualization emphasizes disparities in HeS capabilities across clusters. Source: Authors’ analysis based on GHSI 2021 data.

Based on the final Entropy-CoCoSo assessment scores, EU countries were grouped into three health security performance clusters for both the sub-assessment years and the entire analyzed period ( Table 12, Figure 6). These clusters range from “High” to “Dangerous,” providing a nuanced picture of health security preparedness across the EU.

**Table 12.** Health security performance clusters of the EU-27 countries.

Cluster (Level)	Case - 2019	Case - 2021	The whole period (2017-2021)
**1 (High)**	Belgium, Bulgaria, Denmark, Finland, France, Germany, Latvia, Netherlands, Slovenia, Spain, and Sweden	Belgium, Bulgaria, Denmark, Finland, France, Germany, Latvia, Netherlands, Slovenia, Spain, Sweden, and Lithuania	Belgium, Bulgaria, Denmark, Finland, France, Germany, Latvia, Netherlands, Slovenia, Spain, Sweden, and Lithuania
**2 (Intermediate)**	Austria, Croatia, Czech Republic, Estonia, Greece, Hungary, Ireland, Italy, Lithuania, Poland, Portugal, and Slovakia	Austria, Croatia, Czech Republic, Estonia, Greece, Hungary, Ireland, Italy, Poland, Portugal, and Slovakia	Austria, Croatia, Czech Republic, Estonia, Greece, Hungary, Ireland, Italy, Poland, Portugal, and Slovakia
**3 (Dangerous)**	Cyprus, Luxembourg, Malta, and Romania.	Cyprus, Luxembourg, Malta, and Romania	Cyprus, Luxembourg, Malta, and Romania

**Table 12** categorizes countries into three clusters (High, Intermediate, Dangerous) using K-means clustering of composite Entropy-CoCoSo scores. Cluster assignments reflect temporal stability or shifts in health security capabilities across the study period.

***4.4.1 Bridging gaps in EU health security: Strategic interventions for cluster-specific risks***

Clustering is crucial for revealing patterns in data, enabling targeted strategies and informed decisions. ^50– 53^ An analysis of EU health security scores ( Figure 6) reveals stark disparities across clusters, underscoring the urgent need for targeted, collaborative interventions.

In prevention, Cluster 1 leads significantly in antimicrobial resistance management (AMR: 82.98), while Cluster 3 (Cyprus, Luxembourg, Malta, and Romania) trails far behind (68.75), posing a substantial threat to regional biosecurity. Zoonotic disease control followed a similar pattern, with Cluster 1 performing the strongest (42.82) and Cluster 3 critically weak (23.58), exposing vulnerabilities to pandemic preparedness. Biosecurity scores were similarly stratified, with Cluster 1 achieving 56.23 and Cluster 3 lagging at 41.00. Biosafety measures revealed an even steeper drop, plunging from Cluster 1 (75.00) to a critical low in Cluster 3 (31.25), highlighting systemic deficiencies in safety management. ^9^ Dual-use research and the culture of responsible science are nearly absent in Clusters 2 and 3 (0.00 in both), confined almost exclusively to Cluster 1 (13.88), reflecting the widespread neglect of responsible scientific governance. Interestingly, Cluster 2 outperformed slightly in immunization (79.55) compared to Cluster 1 (78.13), despite its broader systemic weaknesses, while Cluster 3 lagged significantly (59.38), suggesting fragmented prioritization of health initiatives.

Turning to detection and reporting, Cluster 1 once again dominated across laboratory system strength (82.29), surveillance reporting (65.63), and data transparency (88.75), while Cluster 3 consistently recorded the lowest scores, notably in real-time surveillance (9.38) and laboratory supply chains (0.00), leaving critical gaps in early warning capabilities. Although Cluster 2 performs moderately well in laboratory systems, it dramatically underperforms in laboratory supply chain readiness (4.55), which is a potential bottleneck in emergency response.

Emergency response capacities also showed major disparities: Cluster 1 demonstrated stronger preparedness planning (53.12) and operational linkages between public health and security authorities (79.17), whereas Cluster 3 scores were critically low in preparedness (12.50) and response operations (8.33). Despite moderate performance in access to communication infrastructure across all clusters, risk communication effectiveness weakens progressively from Cluster 1 (71.00) to Cluster 3 (51.05).

By shifting focus to health systems, contrasts grow starkers. Infection control practices stand out, with both Cluster 1 and Cluster 2 achieving full scores (100.00), while Cluster 3 dramatically underperforms (50.00). However, substantial weaknesses remained in communication with healthcare workers during emergencies, where Cluster 3 scored zero and Cluster 2 trailed significantly (9.09). Medical countermeasure testing struggles with inconsistencies across clusters: Cluster 1 achieves a relatively strong capacity (71.88), Cluster 2 (65.91), and Cluster 3 (62.50) show noticeable declines, reflecting uneven readiness to approve and deploy new treatments during health crises. More critically, Cluster 3 scored zero in both infection control communication and medical countermeasure deployment, a failure that amplifies systemic vulnerabilities and heightens regional risk during public health emergencies.

In the international commitment domain, strong scores are observed across clusters for cross-border agreements and international commitments (above 98%), suggesting that regional cooperation frameworks are robust. Nevertheless, financing (Cluster 3:13.56) and participation in international evaluation mechanisms (Cluster 3:0.00 for JEE and PVS) highlight major gaps in sustainable commitment to global health security.

Finally, in terms of risk environment factors, all clusters show relatively balanced performance, although Cluster 1 maintains a slightly higher resilience in political security (79.04) and infrastructure adequacy (82.64). Public health vulnerabilities remain concerning across all clusters but are least severe in Cluster 1 (70.95) and most critical in Cluster 3 (67.20). Collectively, these trends revealed a fragmented landscape. High performers, such as Cluster 1, cannot offset systemic failures in Clusters 2 and 3 without EU-wide collaboration. To bridge these gaps, policymakers must prioritize dual-use research governance, laboratory infrastructure upgrades, and standardized crisis drills, leveraging cluster strengths while addressing their unique vulnerabilities. Only through such cohesive action can disparities evolve into unified resilience

***4.4.2 Mapping EU health security: Strengths, gaps, and policy implications across clusters***

The EU’s health security clustering reveals a complex interplay of strengths and vulnerabilities across its member states. High-performing nations—such as Belgium, Bulgaria, Denmark, Finland, France, Germany, Latvia, the Netherlands, Slovenia, Spain, Sweden, and Lithuania—lead in antimicrobial resistance management, laboratory system strength, and emergency preparedness, serving as benchmarks for global health security. However, gaps in biosafety governance, dual-use research policies, and financing persist even among these top performers, underscoring the need for continuous improvement. Notably, the inclusion of countries spanning diverse economic and political backgrounds in this high-performing cluster demonstrates that effective health strategies transcend traditional divides, advocating for cross-regional knowledge-sharing to bolster resilience.

Similarly, “intermediate cluster” countries—Austria, Croatia, the Czech Republic, Estonia, Greece, Hungary, Ireland, Italy, Poland, Portugal, and Slovakia—excel in immunization coverage, infection control practices, and cross-border collaboration but face critical risks in laboratory supply chain management, surveillance responsiveness, and emergency communication—patterns that mirror systemic gaps across this cluster. Their moderate performance highlights both opportunities for strategic growth and urgent areas requiring targeted support.

Most critically, Cluster 3 (Cyprus, Luxembourg, Malta, and Romania) exemplifies systemic fragility, with severe deficiencies in biosafety, real-time surveillance, medical countermeasure deployment, and dual-use research governance. These vulnerabilities not only jeopardize national preparedness but also create weak links in the EU’s broader health security network.

These disparities highlight interconnected challenges: gaps in surveillance infrastructure, health workforce capacity, and medical countermeasure readiness plague multiple clusters, demanding EU-wide standardization and policy coherence. To bridge these gaps, policymakers must prioritize centralized funding mechanisms and mentorship programs that leverage the expertise of high-performing states. ^5, 6^ By fostering equity in resource allocation, promoting cross-border technical support, and building a collaborative governance model, the EU can transform its mosaic of strengths and weaknesses into a unified, resilient defence against emerging health threats.

### 4.5 Implication of study

This study offers important insights for policymakers, researchers, and international health organizations by introducing the Entropy-CoCoSo-K-means framework to address limitations in traditional global indices such as the GHSI. This integrated method allows for a more nuanced and context-sensitive assessment of health security across EU member states. The identification of three distinct performance clusters—ranging from high to dangerous—provides a clear roadmap for differentiated, targeted interventions. High-performing countries such as Finland and Germany can serve as regional benchmarks, facilitating cross-border collaboration and capacity building. In contrast, underperforming countries like Cyprus and Malta require urgent investment in laboratory infrastructure, biosafety, and emergency response mechanisms to bridge critical preparedness gaps. The study also reveals how intra-EU disparities in health system resilience, detection capacity, and rapid response reflect both structural inequalities and varied national priorities. These findings emphasize the need for coordinated surveillance harmonization and investment in shared infrastructure, particularly through EU initiatives like HERA and the European Health Union (EHU).

Comparative insights from Africa and the Eastern Mediterranean Region (EMR) further underscore the importance of regionalized strategies. While the EU emphasizes detection and response systems, African health systems prioritize prevention, and EMR countries place greater weight on the resilience of their health infrastructure. These contrasts point to mutual learning opportunities. For instance, the EU can support Africa in strengthening surveillance and rapid response capabilities, while benefiting from African and EMR expertise in community-based public health strategies and adaptive responses under resource constraints. Moreover, the observed recalibrations between 2017 and 2021 reflect a broader post-pandemic shift in health security priorities—urging leaders to balance immediate crisis response with long-term structural resilience. By aligning with EU-wide strategies such as the EHU and HERA, this study contributes a robust, metrics-driven model to guide equitable and sustainable health security development across the region.

### 4.6 Comparative contributions of this study

This research significantly advances the field of health security evaluation by overcoming the limitations of mono-dimensional assessments such as the Global Health Security Index (GHSI). ^1^ Unlike prior studies that rely solely on ranking countries by composite scores, this work employs a more flexible and informative methodology—entropy-based multi-criteria decision-making (MCDM) integrated with K-means clustering—to better account for contextual variability. Compared to existing approaches, ^1, 9, 16^ our framework captures intra-EU disparities more dynamically and identifies previously overlooked vulnerabilities, such as deficiencies in dual-use research governance and biosecurity systems in smaller EU states like Cyprus and Malta. ^9^

A major contribution of this study lies in the formulation of strategic cross-cluster recommendations to guide EU-wide resilience-building. For Cluster 1 countries (e.g., Finland, Germany), the recommendation is to serve as technical mentors in EU preparedness exercises and lead policy innovation in biosafety and dual-use research governance. For Cluster 2 countries (e.g., Ireland, Slovakia), investment in laboratory infrastructure and crisis communication—possibly supported through EU recovery funding—is critical to addressing performance bottlenecks. ^47^ Cluster 3 countries (e.g., Cyprus, Malta) must prioritize structural reforms and external support through HERA-led interventions, international partnerships, and regional simulation drills to build core capabilities.

These findings support the mission and structure of both HERA and the EHU by offering a data-driven, actionable framework for resource allocation, cross-border solidarity, and long-term preparedness. They also contribute empirical guidance for harmonizing national health systems under supranational governance models. ^11^ Furthermore, lessons from Africa and EMR contexts reinforce the need for adaptive, bottom-up health strategies. African systems offer replicable community-based prevention models, while EMR countries exemplify resilience under crisis—both of which are instructive for refining EU strategies. ^4, 38^ In sum, this study not only fills a methodological gap but also elevates the policy relevance of health security analytics in a post-COVID global landscape.

### 4.7 Limitations and future work

Despite its contributions, this study has several limitations that should be acknowledged. First, the temporal coverage (2017–2021) excludes more recent developments, such as the emergence of Omicron sub variants and key EU reforms like the European Health Data Space (EHDS). ^54^ Extending the analysis to include post-2021 data would provide a more current picture of evolving health security dynamics. Second, the exclusive use of quantitative metrics may miss qualitative dimensions such as political leadership, institutional trust, and public engagement, which are critical for understanding the real-world effectiveness of health systems. Future research should integrate qualitative methods, including stakeholder interviews or case studies, to complement the MCDM framework.

Expanding the Entropy-CoCoSo-K-means approach to subnational or non-EU regions would also help assess its scalability and identify micro-level disparities. Moreover, in-depth studies are needed to explore the causal drivers behind cluster-specific weaknesses—such as differences in governance models, socioeconomic inequality, or healthcare workforce capacity. Longitudinal research tracking targeted interventions in low-performing states like Malta and Cyprus could evaluate whether policy changes lead to measurable improvements. Lastly, deeper inquiry into the root causes of divergent health security priorities between regions like the EU and Africa—whether geopolitical, structural, or fiscal—would further enrich the comparative analysis and enhance the applicability of the framework in broader international contexts.

## 5. Conclusion

This study systematically evaluated health security patterns across the European Union using a hybrid Entropy-CoCoSo and K-means clustering approach, addressing critical gaps in existing supranational analyses. By analyzing six GHSI indicators, the research definitively identified detection and reporting (0.36–0.37 weight) and rapid response (0.20–0.21) as the most critical drivers of disparities in health security performance among EU member states. Temporal trends revealed significant post-pandemic shifts, including growing divergences in health system resilience and persistent stagnation in compliance with international norms, which demand urgent policy attention. The clustering of countries into three distinct tiers (high to dangerous) starkly exposed systemic vulnerabilities in lower-tier nations, characterized by fragmented surveillance networks, chronic underfunding of emergency protocols, and a lack of robust biosecurity measures.

The hybrid framework introduced here significantly advances methodological rigor in health security research, offering a robust and replicable model for regional benchmarking and comparative analysis. Cross-regional comparisons illuminated context-specific priorities, with the EU’s emphasis on detection and rapid response contrasting sharply with Africa’s prevention-centric strategies, highlighting the need for tailored interventions. These findings underscore the imperative for collaborative, tiered interventions—leveraging high-performing clusters as best-practice models while strategically channelling resources and technical assistance to vulnerable states to address their unique deficits.

For EU policymakers, this study provides a roadmap for actionable strategies: strengthening and harmonizing data-sharing protocols under the European Health Union (EHU), prioritizing targeted infrastructure investments in critically vulnerable member states like Cyprus, Luxembourg, Malta, and Romania, and institutionalizing dynamic, continuous monitoring to track progress and adapt to evolving threats.

However, this study is not without its limitations. The reliance on available data may introduce biases, and the entropy method, while robust, may not capture all nuances of health security dynamics. Future research should explore longitudinal studies that incorporate qualitative assessments to enrich quantitative findings. Additionally, expanding the analysis to include other regions could enhance the understanding of global health security patterns.

By effectively bridging methodological and operational gaps, this work contributes to a more resilient, equitable, and proactive health security landscape, not only within the EU but also as a model for other regions. Future efforts must build upon these insights, ensuring that preparedness strategies evolve in tandem with emerging global health threats and are underpinned by robust, data-driven frameworks.

## Ethics and consent

No Ethical approval or consent needed.

## Declaration of generative AI and AI-assisted technologies in the writing process

During the preparation of this work the author(s) used [DeepSeek v3, Paperpal, Quillbot, and ChatGPT] for language refinement and structure. After using this tools, the author(s) reviewed and edited the content as needed and take(s) full responsibility for the content of the publication.

## Data Availability

The data supporting the findings of this study are publicly available and can be accessed through the following repository (Global Health Security Index, Global Health Security Index Data Model and Report (2021), at https://ghsindex.org/report-model/). ^37^ Figshare: Unveiling Health Security Patterns in the European Union_Supplementary Document. Doi: https://doi.org/10.6084/m9.figshare.28898594.v1. ^42^ The project contains the following underlying data: Unveiling Health Security Patterns in the European Union_Supplementary Document.xlsx. All data, and processing results related to this study are presented in this file. This file integrates the processes of weighting, ranking, and clustering analyses into a single Excel-based tool, offering a comprehensive framework for analysis the Health Security in EU Countries. This source also includes the values behind the results reported. This file also includes the values behind the measures reported in all analysis and discussion sections, as well as the values used to construct tables and figures. This supplementary resource also provides detailed support for replicating the study’s methods and results. This data are publicly available and can be accessed through the following repository ( https://figshare.com/articles/dataset/Unveiling_Health_Security_Patterns_in_the_European_Union_Supplementary_Document/28898594) and archived via [ https://doi.org/10.6084/m9.figshare.28898594.v1]. ^42^ Data are available under the terms of the Creative Commons Attribution 4.0 International license (CC-BY 4.0).
