# Supplementary material for: Unveiling Health Security Patterns in the European Union through a Hybrid Entropy-CoCoSo and K-Means Clustering Framework
Source: F1000Res. 2025 Jun 19;14:600. [Version 1] doi: 10.12688/f1000research.166187.1 (PMC12359129; doi:10.12688/f1000research.166187.1)
Supplement: Peer review history

# Reviewer report: Reviewer response for version 1

**DOI:** 10.5256/f1000research.183140.r393598

**Published:** 2025-08-13

**Authors:** Assoc. Prof. Khalil Hamdi Al-Shqeerat

**Affiliations:** 1 Qassim University, Buraydah, Al Qassim, Saudi Arabia

Manuscript "Unveiling Health Security Patterns in the European Union through a Hybrid Entropy-CoCoSo and K-Means Clustering Framework", Naser A. A.,et al.

 This manuscript is a methodologically robust and timely addition to the health security research. It offers a well-designed framework for assessing pandemic preparedness among EU member states from a data-driven, context-sensitive perspective. The integration of Entropy-CoCoSo and K-means provides a strategically balanced approach that combines analytical rigor with practical relevance. The inclusion of cross-regional comparisons with Africa and the EMR enhances the global applicability of this study. With clear exposition, comprehensive data, and practical insights, this study is highly pertinent to both scholars and policymakers. Its capacity to translate complex methods into replicable, actionable guidance solidifies its value as a reference for health ministries and regional preparedness agencies. Although the manuscript is already well developed, a few minor adjustments could further enhance its clarity and overall polish.

  Introduction: When discussing the EHU and HERA, we briefly compare their structures with the WHO frameworks to underscore the distinctive governance complexity in the EU. Additionally, please expand the final paragraph of the Introduction to clearly define the manuscript's core contributions and how they directly address the stated research questions.

 Methodology: Provide concise justifications for choosing CoCoSo over other MCDM techniques, emphasizing its adaptability, interpretability, and empirical success in similar domains. 

 Results: Following Table 10, a summary paragraph that highlights the countries experiencing the most significant rank shifts, both upward and downward, and comments on any unexpected changes in tier positions. 

 Discussion: This section emphasizes a few surprising findings, such as Bulgaria’s strong performance, and contextualizes them in relation to known health system characteristics or reforms. 

 Conclusion: Conclude with a forward-looking paragraph that briefly reflects on the limitations (even if reiterated in Section 4.6), outlines directions for future research, and advocates regional adoption or mentorship programs to foster solidarity across clusters.

Is the work clearly and accurately presented and does it cite the current literature?

Yes

If applicable, is the statistical analysis and its interpretation appropriate?

Yes

Are all the source data underlying the results available to ensure full reproducibility?

Yes

Is the study design appropriate and is the work technically sound?

Yes

Are the conclusions drawn adequately supported by the results?

Yes

Are sufficient details of methods and analysis provided to allow replication by others?

Yes

Reviewer Expertise:

Security, Wireless Sensors Networks, Cloud Computing, Artificial Intelligence.

I confirm that I have read this submission and believe that I have an appropriate level of expertise to confirm that it is of an acceptable scientific standard.

# Reviewer report: Reviewer response for version 1

**DOI:** 10.5256/f1000research.183140.r393596

**Published:** 2025-07-29

**Authors:** Assis. Prof.Nail Adeeb Ali Abdu

**Affiliations:** 1 University of Science and Technology, Yeman, Saudi Arabia; 2 University of Science and Technology, Aden, Yemen

This manuscript makes a significant and timely contribution to the field of health security analytics. By integrating Entropy-CoCoSo and K-means clustering, it introduces a novel methodological approach that is both rigorous and well-executed. The paper demonstrates exceptional analytical depth, particularly in its cross-regional comparison of health security priorities across the EU, Africa, and the Eastern Mediterranean Region (EMR). The clarity in methodological articulation and the robust data visualization throughout the study enhance its impact, making the findings highly accessible to both policymakers and academic audiences. Furthermore, the authors are commended for their comprehensive documentation of each computational stage, the thoughtful discussion of entropy dynamics across regions, and the practical implications of the clustering results. The policy relevance—especially concerning European Health Union priorities—is clearly articulated, and the dataset employed is transparent and replicable. Nonetheless, a few revisions could further enhance the manuscript: 

 1. Abstract: Consider quantifying key results (e.g., entropy weights or number of countries per cluster) to underscore the analytical depth of your framework. 

 2. Introduction: The comparative regional framing (EU vs. Africa and EMR) is excellent. You might elevate this with a sentence summarizing why these two regions were selected for benchmark comparison. 

 3. Methods: Clarify the rationale for comparing 2019, 2021, and aggregated (2017–2021) datasets. A sentence explaining the added value of this temporal analysis would solidify methodological transparency. 

 4. Discussion: When discussing shifts in entropy weights, it could be beneficial to briefly connect these to real-world policy developments in Europe (e.g., HERA’s activation post-2021). 

 5. Conclusion: Consider explicitly stating how your approach could be adapted by health policymakers or by global health institutions such as WHO or ECDC.

 I recommend accepting the paper with minor revisions to address the comments outlined above (Approved).

Is the work clearly and accurately presented and does it cite the current literature?

Yes

If applicable, is the statistical analysis and its interpretation appropriate?

Not applicable

Are all the source data underlying the results available to ensure full reproducibility?

Yes

Is the study design appropriate and is the work technically sound?

Yes

Are the conclusions drawn adequately supported by the results?

Yes

Are sufficient details of methods and analysis provided to allow replication by others?

Yes

Reviewer Expertise:

Machine learning, health information systems, Information security, Blockchain.

I confirm that I have read this submission and believe that I have an appropriate level of expertise to confirm that it is of an acceptable scientific standard.
